# Heart recipient outcomes following transplantation of donor hearts with impaired versus normal function: a study protocol for IMPROVED Heart, a prospective multicentre observational study

**DOI:** 10.1136/bmjopen-2025-111146

**Published:** 2026-07-10

**Authors:** Rana Doueh, Göran Dellgren, Lisa Ternström, Lisa Hård Af Segerstad, Pia Löwhagen Hendén, Jonatan Oras

**Affiliations:** 1 Anesthesiology and Intensive Care Medicine, University of Gothenburg Institute of Clinical Sciences, Gothenburg, Sweden; 2 Department of Cardiothoracic Surgery, Sahlgrenska University Hospital, Gothenburg, Sweden; 3 Department of Transplantation Surgery, Sahlgrenska University Hospital, Gothenburg, Sweden

**Keywords:** Echocardiography, Heart failure, Adult intensive & critical care, Observational Study, Cardiothoracic surgery, Transplant surgery

## Abstract

**Background:**

Cardiac transplantation remains the most effective treatment for patients with end-stage heart failure, but its use is limited by donor organ shortage. Expanding donor acceptance criteria may increase the availability of transplantable hearts. Left ventricular dysfunction due to neurogenic stunned myocardium is common among organ donors and is characterised by rapid functional and structural recovery. The IMPROVED Heart (IMPact of donor heart function on Recipient Outcomes: a prospectiVE observational study to increase the utilisation of Donor Hearts) study aims to increase the number of heart transplantations by systematically evaluating and using donor hearts with temporarily impaired function.

**Methods and analysis:**

IMPROVED Heart is a prospective, multicentre observational study. The primary objective is to determine whether recipients of donor hearts with regional wall motion abnormalities and/or mild to moderate global left ventricular dysfunction have outcomes comparable to recipients of hearts with normal function. Cardiac function in potential heart donors is assessed systematically using repeated echocardiography in addition to cardiac biomarkers and clinical data. Heart transplant recipients are managed according to routine clinical practice. Based on a non-inferiority power calculation, 445 transplanted hearts—including at least 89 with impaired function—are required. The study started enrolling in 2022.

**Ethics and dissemination:**

The study was approved by the Swedish Ethical Review Authority in March 2020 (Dnr 2019-06229). Study findings will be presented at scientific meetings and published in international peer-reviewed journals.

**Trial registration number:**

NCT04393181

**STRENGTHS AND LIMITATIONS OF THIS STUDY**

- First prospective, multicentre study to systematically evaluate donor hearts with impaired function and their use.
- Adequately powered for a non-inferiority analysis of hard endpoints.
- Standardised protocol for donor echocardiographic and clinical assessment improves consistency and supports objective decision-making.
- Use of routine clinical follow-up and national registry data ensures high data completeness and enables long-term outcome assessment.
- Non-randomised design may introduce confounding, particularly related to clinical decision-making by transplant surgeons.

## Introduction

### Background and rationale

 Left ventricular dysfunction among organ donors is a common condition and is seen in 15%–24% of donors after brain death.^1 2^ There is evidence supporting that dysfunction after brain death is highly reversible,^3^,^5^ yet the use of these hearts is scarce.^6^ Despite advancements in pharmacological therapy and mechanical circulatory support, cardiac transplantation continues to be the most effective treatment for end-stage heart failure.^7^ Transplantation rates are, however, limited due to organ shortage, which is worsened by the stringent selection criteria outlined in current guidelines.^8 9^ With an increasing supply–demand mismatch, refusing heart donor candidates contributes to prolonged waiting list times, highlighting the urgent need for the adoption of extended donor criteria.

In organ donors, a catecholamine storm is triggered due to brain herniation and the development of brain death.^10 11^ The catecholamine-rich environment becomes toxic for the heart, thus leading to the development of acute heart failure.^12^,^17^ However, a key feature of this catecholamine-induced acute heart failure is its rapid reversibility, with cardiac function typically normalising within hours to days,^15^,^18^ including a histopathological recovery and normalisation of biomolecular signalling.^12 15^ In addition to organ donors having a primary intracranial event, reversible acute heart failure is also seen in donors with brain death due to hypoxic/anoxic brain injuries following cardiac arrest.^19 20^

There is an ongoing debate within the field of heart transplantation whether donor hearts with temporarily impaired function should be used or not, and clinical practise differs substantially between centres. The 2010 guidelines from the International Society for Heart and Lung Transplantation (ISHLT) advise against the use of donor hearts with regional wall motion abnormalities (RWMA) or left ventricular ejection fraction (LVEF) <40% despite haemodynamic optimisation with inotropic support.^8^ This recommendation to not use hearts with RWMA has inadequate scientific support, based on a retrospective study published over 30 years ago that did not report that RWMA was associated with worse outcomes.^21^ Despite the limited evidence base, this guideline has had a notable influence on clinical decision-making regarding heart acceptance for transplantation.^22^ Several retrospective studies from different transplant centres have since reported that both short- and long-term outcomes are not adversely affected in recipients of donor hearts with transient dysfunction, or have recovered from dysfunction, at the time of transplantation.^123^,^25^

In a retrospective analysis conducted by our research group, we evaluated all potential heart donors over a 10-year period.^26^ Left ventricular dysfunction was identified in 24% of donors, of which 29% were subsequently used for transplantation. Among recipients who received hearts with pretransplant dysfunction (n=42), there was no observed increase in the risk of adverse outcomes, including mortality or need for retransplantation, when compared with recipients of hearts with normal function. Furthermore, short-term clinical outcomes—such as intensive care unit (ICU) length of stay, requirement for advanced haemodynamic support, postoperative dialysis and incidence of acute rejection—did not differ significantly between the groups. Notably, cardiac function recovered rapidly following transplantation, with both groups demonstrating similar LVEFs within a few days postoperatively, contributing to the accumulating evidence indicating that existing donor heart exclusion criteria may warrant re-evaluation.

Given the ongoing shortage of donor hearts, discussions around strategies to expand donor eligibility criteria remain highly relevant and valuable. The most recent evidence-based guidelines, developed by an international panel of experts following an extensive literature review, address the use of donor hearts with low ventricular ejection fraction.^27^ While these recommendations align with existing guidelines, they support the use of such hearts under specific conditions. Nonetheless, prospective studies evaluating outcomes following transplantation of hearts with functional impairment are lacking and are critically needed to inform future clinical practice.

The aim of this manuscript is to describe the design of the IMPROVED Heart study in detail and to inform the research community in this field, thereby contributing to transparency and reproducibility.

### Aim and objectives

**Figure 1:**
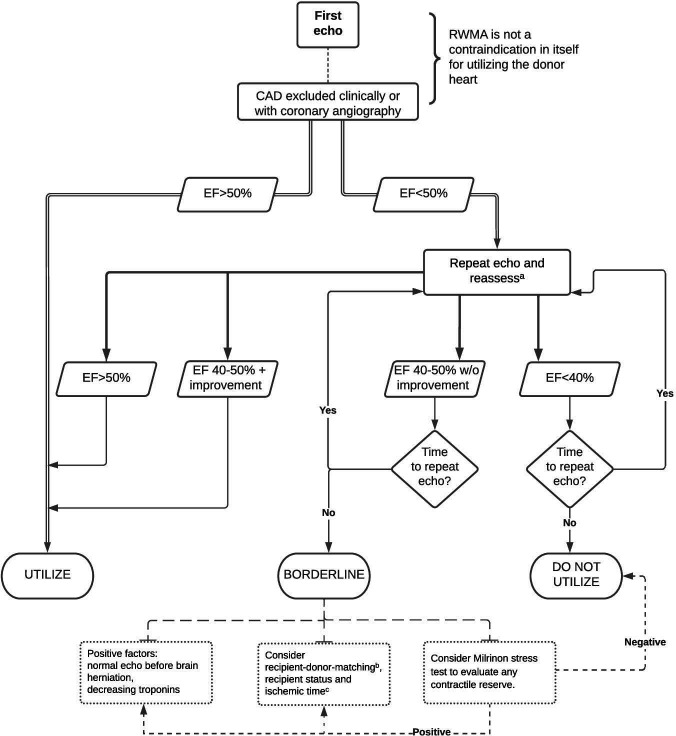
Evaluation protocol for donor heart function. ^a^Preferably made at least 6 hours apart. ^b^*Donor age*: <45 years preferable; 45–50 years if ischaemic time <4 hours; >55 years only if survival benefit exceeds the decrement in early heart transplant survival. *Donor size*: if body weight in donor is not ≥30% less than recipient's, it is uniformly safe; if female donor, use with caution if donor body weight is ≥20% less than male recipient donor weight; donor comorbidities and cause of death. ^c^Ischaemic time should be ≤4 hours; if greater, other factors in donor and recipient should be ideal. CAD, coronary artery disease; EF, ejection fraction; RWMA, regional wall motion abnormalities.(^1^The International Society of Heart and Lung Transplantation Guidelines for the care of heart transplant recipients. *J Heart Lung Transplant* 2010;29:914–56).

The aim of IMPROVED Heart is to increase donor heart utilisation through extended use of donor hearts with temporarily impaired function. Additionally, the study seeks to develop and validate an evidence-based protocol for donor heart evaluation (figure 1). The study also aims to improve the understanding of cardiac dysfunction in organ donors through analysis of clinical data and examination of tissue samples.

It is estimated that the systematic evaluation and inclusion of donor hearts with temporarily impaired function may increase the number of available donor hearts by approximately 20%–30%. In practical terms, this could potentially reduce waiting times for transplantation and may allow for expanded criteria in the selection of heart transplant candidates and thereby increase the number of transplantations.

#### Primary objective

The primary objective is to determine whether recipients of donor hearts exhibiting RWMA and/or mild to moderate globally reduced cardiac function have the same outcomes as recipients of hearts with normal function.

#### Secondary objective

The secondary research questions include:

Does cardiac function differ postoperatively in a recipient who has received a donor heart with a functional impairment compared with a recipient who has received a donor heart with normal function?

With a systematic examination of hearts with impaired function, how many of them recover and by how much does the number of heart transplantations increase? Furthermore, does this affect waiting list time?

Can it be predicted early on which donor hearts will improve function during the donor process using clinical data, echocardiographic imaging and cardiac biomarkers?

What mechanisms underlie cardiac dysfunction in potential heart donors, as determined by comparative histological and biochemical analyses of dysfunctional versus non-dysfunctional donor hearts?

## Methods

### Study design

IMPROVED Heart is a prospective multicentre observational study.

**Figure 2:**
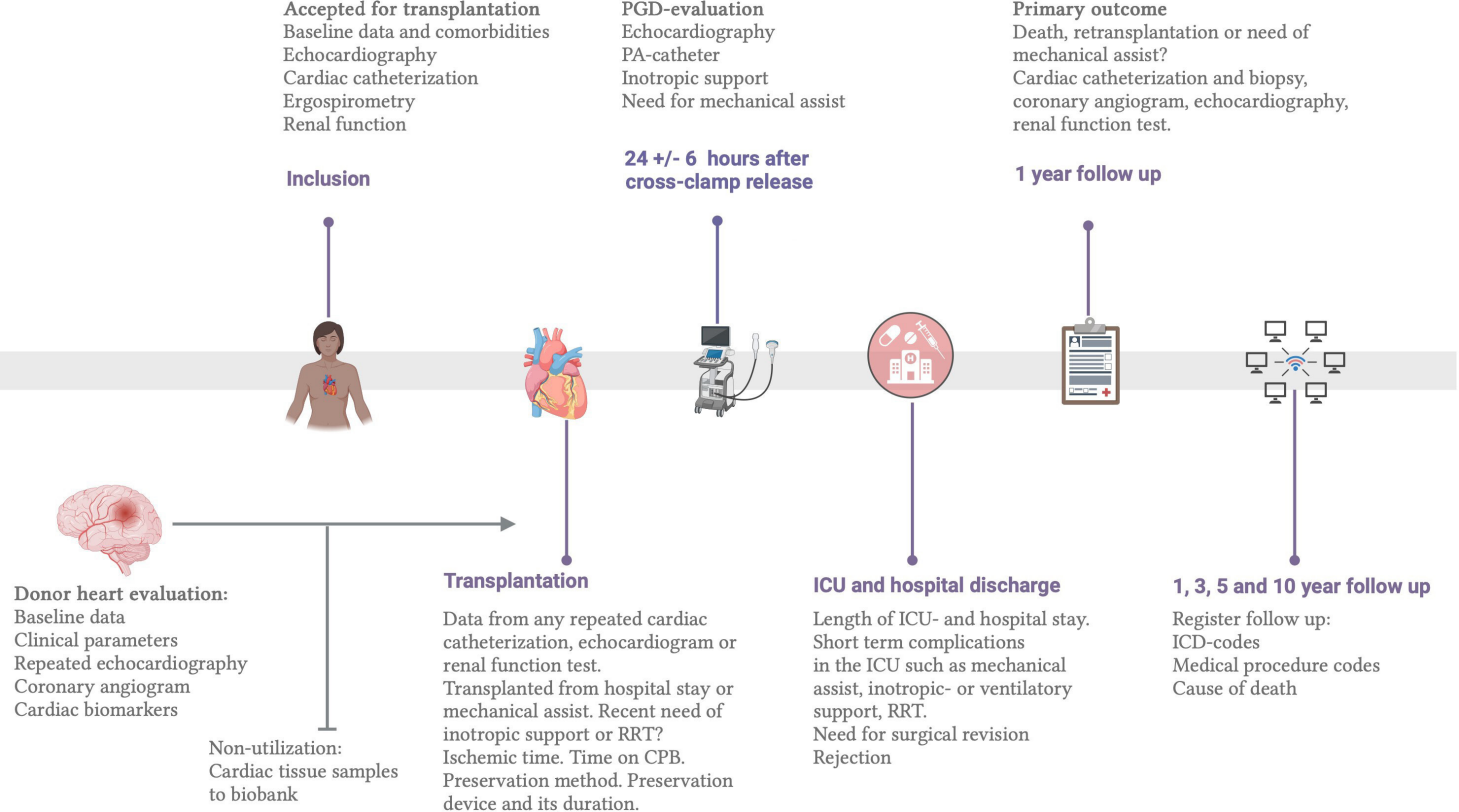
Study overview and data collection timeline. CPB, cardiopulmonary bypass; ICD, International Statistical Classification of Diseases and Related Health Problems; ICU, intensive care unit; PA-catheter, pulmonary artery catheter; PGD, primary graft dysfunction; RRT, renal replacement therapy.

Donor cardiac function is assessed systematically using a predefined evaluation protocol as seen in figure 1. When the donor heart is accepted for transplantation, recipient outcomes are recorded at predefined time points according to figure 2.

### Methodological considerations

Participation in the study will not influence organ allocation; the decision is always made by the thoracic surgeon based on standard donor–recipient matching. Although in theory, patients could be randomised to two separate waiting lists—one accepting donor hearts with dysfunction and one excluding them. However, such a design is currently not feasible in the Scandinavian context. The relatively small number of patients on transplant waiting lists would make donor–recipient matching impractical, particularly given the anticipated high rate of crossover between the groups.

### Study setting

In Sweden, the two centres performing heart transplantations, Sahlgrenska University Hospital in Gothenburg and Skåne University Hospital in Lund, are participating in the study. In addition, Helsinki University Hospital in Finland is included as an active recruitment centre, contributing to heart recipient enrolment and donor data. Additional centres across Scandinavia are planned to be recruited to increase inclusion capacity.

The largest ICUs in Sweden include potential heart donors and provide additional clinical information in more detail. These centres care for approximately 60% of all potential donors in Sweden, and more centres are currently being included.

For the remainder of donor hearts used, donor data are available through the Scandia transplant register, where donor data are registered prospectively during the donation process.

### Eligibility criteria

#### Donor

Inclusion criteria:

To be considered eligible for inclusion in the study, potential donors must meet the following criteria:

- Aged between 18 and 70 years.
- A decision to transition to end-of-life care due to futile prognosis.
- Being positive for organ donation according to national guidelines and legislation.
- The donation/transplant centre has been contacted regarding potential organ procurement.

The donor does not yet have to be declared brain dead at the time of inclusion. Currently, only heart donation following brain death is being investigated; this may be revised should clinical practice in participating countries change.

#### Recipient

Inclusion criteria:

Recipients must meet all the following criteria to be eligible for inclusion in the study.

- Approved for heart transplantation at a participating transplantation centre.
- Has provided written informed consent to participate in the study.
- Aged 18 years or older.

Exclusion criteria:

- Mental inability, reluctance or language difficulties that result in difficulty understanding the meaning of study participation.
- Another study, where the patient is included, which is not considered compatible with the current study.

### Donor heart assessment

For potential donors who meet all inclusion criteria, an initial transthoracic echocardiographic examination is performed as soon as logistically feasible. Subsequent echocardiographic assessments are conducted in accordance with the evaluation protocol for donor heart function seen in figure 1. The protocol outlines possible clinical scenarios described below.

First, RWMA are not in themselves considered a contraindication for heart transplantation if coronary artery disease is excluded according to clinical guidelines^27^ or with coronary angiography.

- Scenario 1: if the echocardiogram shows normal cardiac function—defined as an LVEF ≥50%—the heart may be accepted for transplantation.
- Scenario 2: if the echocardiogram shows reduced cardiac function—defined as LVEF ≤50%—the examination is repeated. The study protocol recommends a minimum waiting period of 6 hours before reassessment, based on evidence suggesting that left ventricular function can improve by approximately 10% within 12 hours.^26^ A ≥10% increase in LVEF is considered clinically relevant. If left ventricular function improves to an ejection fraction ≥50%, the heart may be accepted for transplantation. If LVEF remains between 40% and 50% but shows improvement compared with the previous examination, the heart is recommended for use. Echocardiographic assessments may be repeated multiple times as clinically appropriate, until the donation process concludes.
- Scenario 3: if the echo shows LVEF 40%–50% without signs of improvement, additional factors are considered such as ischaemic time, donor–recipient matching, and recipient status. Supporting indicators may include previously documented normal cardiac function earlier during the donor’s hospital stay or a decreasing trend in cardiac biomarkers (eg, troponin). A pharmacological stress test, such as with milrinone or dobutamine, may be considered to evaluate any contractile reserve.
- Scenario 4: in cases where LVEF remains ≤40%, with or without evidence of functional improvement on serial echocardiography, the heart cannot be recommended for utilisation in the study setting.

These recommendations are based on the retrospective studies available.^1 18 23 24^ The protocol for systematic evaluation with echocardiographies ensures that cardiac dysfunction that is dynamic and reversible is identified. The final decision to use a donor heart remains with the thoracic surgeons, and there may be additional clinical factors, such as specific donor–recipient matching, that the study protocol cannot measure. In non-utilisation, the causes will be documented in detail as in online supplemental material (S1).

In conjunction with each echocardiographic examination, blood samples for cardiac biomarkers (cardiac troponin I or T, depending on local routine, N-terminal pro-B-type natriuretic peptide) are collected for analysis. Clinical data (eg, mechanical ventilation settings and inotropic support) are also recorded. Additional donor characteristics are documented as outlined in the online supplemental material (S1). A detailed description of donor management regarding monitoring, treatment targets and treatment recommendations is provided as online supplemental material (S2), Swedish National Guidelines for Donor Management.

Hearts not used for transplantation but donated for other medical purposes, such as heart valve retrieval through tissue banks or medical research, will undergo three myocardial punch biopsies, one from the apical myocardial region, one from the interventricular septum and one from the basal segment of the lateral left ventricular wall. The biopsies will be stored in a certified biobank for subsequent analyses and future research in accordance with applicable regulations.

### Participant timeline

A schematic overview of the study schedule and participant time points is shown in figure 2. The complete study protocol is provided as an online supplemental file (S3). No interventions will be introduced for recipients, who will receive standard care in accordance with clinical practice.

Recipient data will be collected prospectively and assessed at six predefined time points. The collected variables are selected to characterise baseline recipient status, perioperative factors, early graft function and short- and long-term transplant outcomes.

1. At acceptance of transplantation (inclusion)—recipient demographics, underlying diagnosis leading to transplantation and relevant comorbidities. Baseline clinical status will include echocardiographic assessment of cardiac function, renal function, ergospirometry and haemodynamic measurements obtained from right heart catheterisation. Information on pretransplant circulatory support, including inotropes and mechanical circulatory support at the time of listing, will also be recorded.
2. At the time of transplantation—recipient clinical status immediately prior to transplantation will be documented, including whether transplantation occurred during ongoing hospitalisation, use of mechanical circulatory assist devices and recent need for inotropic support or renal replacement therapy. Updated clinical assessments available prior to transplantation, including repeated echocardiographic assessment of cardiac function, haemodynamic measurements obtained from right heart catheterisation and renal function measurements, will also be collected. Perioperative variables including ischaemic time, cardiopulmonary bypass duration, preservation strategy, and use and duration of preservation devices.
3. At 24 hours (±6 hours) after cross-clamp release—assessment of early graft function including haemodynamic parameters, inotropic requirements, need for mechanical circulatory support and evaluation of primary graft dysfunction defined according to the ISHLT criteria.^28^
4. At discharge from the ICU—duration of intensive care, need for ongoing circulatory support, renal replacement therapy and other early postoperative complications.
5. At hospital discharge—postoperative complications including surgical reinterventions and rejection episodes.
6. At 1 year follow-up—survival status, retransplantation, need for mechanical circulatory support, haemodynamic measurements, echocardiographic assessment of graft function, haemodynamic measurements obtained from right heart catheterisation and biopsies, coronary angiogram and renal function test.

Additional mortality control will be performed at 3 and 6 months after transplantation.

Long-term complications will be collected through the Swedish National Board of Health and Welfare’s Diseases, the Swedish Thoracic Transplantation Register (STRAX) and Cause of Death Register at 1, 3, 5 and 10 years after heart transplantation.

A complete list of variables collected for both donors and recipients is provided in the online supplemental material (S1), with a list of abbreviations seen in online supplemental material (S4).

### Outcomes

#### Primary variable

The primary outcome is a composite of retransplantation and/or death within 1 year following heart transplantation, or the requirement for mechanical circulatory support at 1 year post-transplantation.

#### Secondary variable

The secondary outcome variables include the following:

- Frequency of left-sided moderate or severe primary graft dysfunction (PGD) or right-sided PGD, defined according to the ISHLT criteria.^28^
- Postoperative complications and need for interventions, including use of mechanical circulatory support (eg, ventricular assist device or intra-aortic balloon pump), prolonged mechanical ventilation, need for dialysis, and dosage and duration of inotropic and vasoactive drug support.
- Risks for other diseases by collecting data from the Swedish National Board of Health and Welfare’s Patient Register, STRAX and the Cause of Death Register. The data that will be requested for all patients are International Statistical Classification of Diseases and Related Health Problems codes and medical procedure codes at 1, 3 and 10 years after transplantation.
- Estimated increase in the number of patients eligible for heart transplantation following systematic evaluation of donor hearts with impaired function.
- Levels of cardiac biomarkers, specific echocardiographic findings and clinical characteristics in donors with recovered versus non-recovered cardiac function during the donation process.
- Presence of specific histopathological changes and protein expression profiles in myocardial tissue from hearts with impaired and normal function, respectively, obtained from donors whose hearts were not used for transplantation but were donated for heart valve retrieval or medical research through tissue banks.

Variables for the primary and secondary outcomes are detailed in table 1.

**Table 1:** Primary and secondary outcomes

Primary outcome	Composite of retransplantation and/or death within 1 year following heart transplantation, or the requirement for mechanical circulatory support at 1 year post-transplantation
Secondary outcome	Frequency of left-sided moderate or severe PGD or right-sided PGD, defined according to the International Society for Heart and Lung Transplantation criteria^28^ Postoperative complications and need for interventions* Risks for other diseases by collecting ICD codes and medical procedure codes from national registers^**^ at 1, 3, 5 and 10 years after transplantation Estimated increase in the number of patients eligible for heart transplantation following systematic evaluation of donor hearts with impaired function Levels of cardiac biomarkers,† specific echocardiographic findings‡ and clinical characteristics in donors§ with recovered versus non-recovered cardiac function during the donation process Presence of specific histopathological changes and protein expression profiles in myocardial tissue from non-used hearts with impaired and normal function, respectively¶

* Use of mechanical circulatory support (eg, ventricular assist device or intra-aortic balloon pump), prolonged mechanical ventilation, need for dialysis, and dosage and duration of inotropic and vasoactive drug support.

† Cardiac troponin (I or T depending on local routine) and NT-proBNP.

‡ Left ventricular function: ejection fraction, velocity time integral, stroke volume, Cardiac Index, regional wall motion abnormalities (segments and grading). Right ventricular function: tricuspid annular plane systolic excursion, right ventricular peak systolic tissue velocity, occurrence of D-sign or dilatation of the right ventricle. Pulmonary pressure: tricuspid regurgitation maximum velocity or systolic pulmonary artery pressure. Other: central venous pressure. Valve disorders (type and grading).

§ Comorbidities, cause of death and mechanism, time from injury to declaration of brain death, need of vasoactive drugs, blood pressure, heart rate, mechanical ventilatory support: fraction of inspired oxygen, positive end expiratory pressure, peak pressure, tidal volume in mL/kg predictive bodyweight, partial pressure of oxygen in arterial blood. Hormonal treatment with methylprednisolone. Coronary angiogram.

¶ Three myocardial punch biopsies (apical region, interventricular septum and basal segment of the lateral left ventricular wall) will be taken from hearts not used for transplantation but donated for other medical purposes, such as heart valve retrieval through tissue banks or medical research. Predefined key analyses include S100A8/A9 and the TLR-4 downstream signalling molecules MyD88, NF-kappaβ, IL-6, IL-1β, TNF-α analysed by ELISA or Western blot. Inflammation and inflammatory cell composition will be analysed with immunohistochemistry.

** Swedish National Board of Health and Welfare’s Patient Register, the Swedish Thoracic Transplantation Register and the Cause of Death Register.

ICD, International Statistical Classification of Diseases and Related Health Problems; IL-6, interleukin 6; NF-kappaβ, nuclear factor kappaβ; NT-proBNP, N-terminal pro-B-type natriuretic peptide; PGD, primary graft dysfunction; TNF-α, tumour necrosis factor alpha.

### Sample size

Power calculations are based on a non-inferiority design. In our retrospective cohort, 1-year survival without death or retransplantation was 90%. The sample size was calculated to exclude a difference of 10 percentage points in the primary outcome, which is considered clinically relevant due to the potential benefits of earlier transplantation and increased transplantation rates. We estimate that 20% of transplantations will involve donor hearts with impaired function. Based on these assumptions, a total of 445 heart transplant recipients are required, including at least 89 recipients of hearts with impaired function. To accommodate uncertainty regarding the true incidence and utilisation rate of donor hearts with dysfunction in a prospective setting, we plan to enrol a total of 500 heart transplant recipients.

For secondary analyses related to donor heart function, data from at least 100 donors with impaired function are required to ensure a representative cohort. Assuming a 25% incidence of cardiac dysfunction among potential donors, at least 400 donor cases are needed. Donor data collection will continue beyond this threshold until the primary objective has been met.

### End of study

The study will conclude once the following two criteria are met:

1. A total of 445 heart transplant recipients have been enrolled.
2. At least 89 of these recipients have received donor hearts with impaired function.

Data collection will continue until both conditions are fulfilled.

The study may be prematurely terminated if there is evidence of a high incidence of serious adverse events potentially related to the donor heart function, or if recruitment targets cannot be achieved within a reasonable timeframe. The decision to terminate the study in that case is taken by the sponsor.

### Patient and public involvement

Patients and members of the public were not involved in the design, conduct or analysis of this study. However, information about the study has been actively disseminated through a patient organisation, clinical meetings, and a dedicated study website to promote transparency and awareness of the ongoing research (www.improvedheart.com).

### Data collection methods

Source data are handled according to Good Clinical Practice (GCP) and the General Data Protection Regulation.

#### Donor

All data will be collected from medical records, databases used in donor management, and clinical charts from the ICU and operating theatre. A case report form (CRF) will be completed at the time of echocardiographic assessment. Participating donor hospitals will compile the collected data and submit it to the coordinating centre in Gothenburg.

Echocardiographic examinations will be performed by accredited operators. If echocardiograms are conducted by other personnel, all recordings will be saved, and a designated protocol will be completed and signed. To ensure consistency, all donor echocardiograms will be reviewed by a senior consultant in echocardiography.

A complete list of study variables is provided in online supplemental material (S1).

#### Recipient

Data will be collected at the transplanting hospitals through review of medical records and clinical charts from the ICU and operating theatre. All postoperative echocardiographic examinations in heart transplant recipients will be centrally reviewed to ensure consistency of interpretation.

A complete list of study variables is provided in the online supplemental material (S1).

### Retention

Heart transplant recipients may withdraw their consent to participate in the IMPROVED Heart study at any time. However, as no study-specific interventions are planned and all follow-up aligns with standard clinical care, the risk of participant non-retention is considered low.

### Data management

Data for each donor and recipient will be entered into an electronic CRF (REDCap) using a two-step verification process by trained research personnel.

Source data documents will be securely stored in a locked room with restricted access in accordance with institutional data protection protocols.

### Statistical methods

#### Statistical analysis

The primary outcome—defined as the composite incidence of death, retransplantation or need for long-term mechanical circulatory support within 1 year post-transplantation—will be analysed using binary logistic regression. The primary explanatory variable will be donor cardiac dysfunction, defined as the presence of RWMA and/or an LVEF <50%.

The predefined adjusted analysis includes relevant covariates identified in previous studies, namely recipient age, donor age, pretransplant mechanical circulatory support and transplantation performed under ‘urgent call’ status. In a secondary adjusted analysis, a data-driven statistical approach will be applied, incorporating covariates found to be significantly associated with the primary outcome.

#### Analysis population and missing data

Patients will be included in the primary outcome analysis only if the following key data are available: assessment of donor cardiac function, information on confounding variables required for multivariable adjustment and measurement of the primary outcome. For secondary outcomes, missing data may be handled through imputation in sensitivity analyses, provided the data are deemed to be missing completely at random.

### Data monitoring

To ensure that the study is conducted in accordance with the protocol and that data collection, documentation and reporting comply with GCP and applicable ethical standards, monitoring will be performed by an independent monitor. Monitoring activities will take place prior to study initiation, throughout the study period and following study completion.

Monitoring will be conducted in accordance with the study’s predefined monitoring plan and is intended to ensure that the rights, safety and well-being of study participants are protected. It will also ensure that the data recorded in the CRFs are complete, accurate and consistent with the corresponding source documents.

#### Interim analysis

An interim analysis will be conducted after 100 patients have been assessed for the primary outcome, and subsequently after every additional 50 patients who reach the 1-year follow-up. The study may be terminated early if there is a confirmed over-representation of the primary outcome—defined as death, retransplantation, or the need for long-term mechanical circulatory support—among recipients of hearts from donors with impaired cardiac function. Termination will require both an absolute difference of ≥10 percentage points in the primary outcome and statistical significance (p<0.05). In addition, an increase in the incidence of serious adverse events (SAEs) of ≥20 percentage points, with statistical significance (p<0.05), may also serve as a criterion for early termination.

### Harms

#### Handling of adverse events

Postoperative care following heart transplantation commonly involves intensive care, including circulatory and respiratory support, and, in some cases, renal replacement therapy. As these treatments are considered part of the standard post-transplant course, they will not be classified as adverse events within the context of this study.

*SAEs* are defined as events that require more invasive interventions or result in significant patient harm, including:

- Requirement for postoperative mechanical circulatory support lasting more than 24 hours after transplantation.
- Death occurring within 28 days of cross-clamp release.
- Other events resulting in prolonged hospitalisation or significant morbidity (eg, stroke with residual deficits) that are attributable, or suspected to be attributable, to donor cardiac function.

Each SAE will be reviewed to assess a potential causal relationship with the cardiac function of the donor heart. The incidence of SAEs will be compared between groups and included in interim analyses.

All SAEs must be reported to the study sponsor using a designated SAE reporting form within 24 hours of the investigator becoming aware of the event.

In the event of an increased incidence of SAEs observed during interim analyses, the study may be subject to early termination.

As the study is non-randomised and unblinded, no independent data safety monitoring board is planned.

### Auditing

No auditing is planned for the study. However, if deemed necessary by the sponsor or regulatory authorities, an independent audit may be conducted during or after the study to ensure compliance with applicable regulations and to verify data integrity.

## Ethics and dissemination

### Research ethics approval

The study protocol, along with the final versions of the informed consent form and participant information materials, has been approved by the Swedish Ethical Review Authority (Etikprövningsmyndigheten, Dnr 2019-06229). For the study site in Helsinki, Finland, ethical approvals were obtained from Helsinki University Hospital Regional Research Ethics Committee (approval date 23 March 2025, HUS 10/2025). Organs and tissue have and will continue to be sourced ethically and not from executed prisoners, prisoners of conscience or other vulnerable groups.

### Protocol amendments

Any modifications to the study protocol or informed consent documents will be submitted to the Swedish Ethical Review Authority, as well as to Helsinki University Hospital Regional Research Ethics Committee, for review and approval in accordance with applicable regulatory requirements.

### Consent or assent

#### Donor

In accordance with the approval granted by the Swedish Ethical Review Authority (Dnr 2019-06229) and Helsinki University Hospital Regional Research Ethics Committee (HUS 10/2025), no informed consent from the donors’ relatives is required. As the study does not register any data prior to the donors’ death, the donors are not classified as research subjects. Furthermore, all investigations are carried out as part of clinical routine, but are now performed in a systematic manner, without introducing any study-specific interventions or discomfort for the donor.

#### Recipient

Written informed consent will be obtained from all participants prior to the initiation of any study-specific procedures. The study will be conducted in accordance with the principles of GCP and the ethical guidelines outlined in the Declaration of Helsinki (2013).

## Access to data

The study sponsor, principal investigator, co-investigators, designated data entry personnel, and the study monitor will have access to the electronic case report forms (e-CRFs), where de-identified data are entered. User rights are role-based, providing access only as needed for specific study responsibilities. Documents containing personal identification numbers and source data will be accessible only to authorized individuals as required for their study responsibilities, in accordance with applicable data protection regulations.

## Ancillary and post-trial care

Research subjects are insured by Swedish Patient Insurance during the study.

## Dissemination policy

Every effort will be made to minimize the time between the completion of data collection and the dissemination of study results. There are no publication restrictions.

Study findings will be presented at local and international scientific meetings and submitted for publication in peer-reviewed international journals, regardless of the outcome.

## Supplementary material

10.1136/bmjopen-2025-111146 online supplemental file 1

10.1136/bmjopen-2025-111146 online supplemental file 2

10.1136/bmjopen-2025-111146 online supplemental file 3

10.1136/bmjopen-2025-111146 online supplemental file 4

10.1136/bmjopen-2025-111146 online supplemental file 5
