# Supplementary material for: Heart recipient outcomes following transplantation of donor hearts with impaired versus normal function: a study protocol for IMPROVED Heart, a prospective multicentre observational study
Source: BMJ Open. 2026 Jul 10;16(7):e111146. doi: 10.1136/bmjopen-2025-111146 (PMC13358299; doi:10.1136/bmjopen-2025-111146)
Supplement: online supplemental file 1 [file bmjopen-16-7-s001.pdf]

Consent form for IMPROVED Heart

IMPact of donor heart function on Recipient Outcomes - a prospectiVE study to increase the utilization of Donor HEARTs

We are asking you to participate in a research study focused on investigating the potential impact of donor heart function on the outcomes of heart transplantation recipients. You are being invited to take part in this study as you are scheduled to undergo a heart transplantation. The study is being conducted at the transplantation units of Sahlgrenska University Hospital and Skåne University Hospital, and it has been reviewed and approved by the Swedish Ethical Review Authority. This document provides information about the study and outlines what participation will involve.

Background and Purpose

Occasionally, organ donors who are otherwise healthy may experience impaired heart function. This dysfunction can manifest in various forms, but it is generally a temporary condition. In these cases, the heart itself is not defective; rather, its function is affected by external factors associated with the donor's brain death. Once the donor's condition stabilizes, heart function typically recovers, often within a few hours to days.
In Sweden, there is significant experience with transplanting such hearts, and our observations indicate that recipients of these hearts experience outcomes comparable to those of recipients with hearts that have not been impaired. However, not all transplant centers worldwide utilize these hearts. Sharing this knowledge could potentially increase the global number of heart transplants by 20-30%.
We are currently conducting a study to determine whether transplantation with a donor heart that has undergone functional impairment affects recipient outcomes.

How is the Study Conducted?

In the study, the donor heart will undergo additional evaluation. However, no further tests will be conducted on you as the heart recipient. Therefore, participation in the study will not require any additional interventions, and you will continue to be monitored according to the current standard protocol for all heart transplant patients. The study will document details about your transplant process and collect data for up to one year following the transplantation. Additionally, we will obtain information from Sweden's national health registers at three, five-, and ten-years post-transplantation to assess any subsequent diseases or procedures.
Your participation in the study will not affect the heart you receive. We ensure that you always recieve the best available organ suited for you.

Advantages and Disadvantages of Participation

The study will not cause any additional exams or discomfort as you will be monitored according to the current standard protocol for heart transplant patients.
While the donor heart will undergo a thorough evaluation, which may benefit the recipient, we cannot guarantee that you will experience a direct benefit from the study. However, the knowledge gained will contribute to improving outcomes for many future heart transplant recipients.

Voluntariness

Your participation in this study is voluntary, and you may withdraw your consent at any time without the need to provide a reason. Withdrawing from the study will not affect the care you receive in any way.
If you wish to withdraw**:** Contact the principal investigator (contact details below).
Collected Information**:** The principal investigator has the right to retain data collected before withdrawal, which will remain part of the study.

Research personnel and contact information

Before deciding, it is important to fully understand what the study involves for you. Should you have any questions or need additional information, please feel free to discuss it with your doctor or the principal investigator listed below.

Principal Investigator:
Dr. Göran Dellgren, Senior Consultant/Professor
Phone: 031-3420000 (switchboard)
Email: goran.dellgren@vgregion.se

Should you have any questions regarding the study, do not hesitate to contact us.

Data Management and Privacy

During the study, we will gather information such as your date of birth, gender, health history (including current and past illnesses), and examination results. This data will be stored in a secure register and processed for research purposes, which is legally justified by its public interest.
Your data will be protected by confidentiality laws, ensuring that unauthorized individuals cannot access the register. All the data will be handled in a way that prevents identification of individual participants during analysis, reporting, or publication.

According to the General Data Protection Regulation (GDPR), you have the right to request information about which personal data is being processed, correct or delete personal data, or request restrictions on processing. If needed, you can contact the Data Protection Officer (DPO).

The Executive Board of Sahlgrenska University Hospital is responsible for the processing of personal data. The Data Protection Officer is tasked with ensuring that your personal data is handled in a lawful and accurate manner. If necessary, the Data Protection Officer can assist you in accessing information about the data that has been recorded and facilitate any required corrections.
The Data Protection Officer can be contacted at the following address:
Sahlgrenska University Hospital, Data Protection Officer, 413 45 Gothenburg.
Phone: +46 31-343 27 15.
Email: sahlgrenska.universitetssjukhuset.dso@vgregion.se.

Complaints regarding personal data processing can be directed to the Swedish Data Protection Authority.

Your personal data will only be used for the purposes stated above. They may only be processed for other purposes if you provide new consent and/or new approval is obtained from the Swedish Ethical Review Authority.

In addition to healthcare personnel, an individual designated by the clinic or an official may review the collected study data alongside your medical records for quality assurance purposes, ensuring the study is conducted properly. These individuals will be required to sign a confidentiality agreement before accessing your medical records. Study data will be retained for a minimum of 10 years following the completion of the study to facilitate any necessary audits. By signing this consent form, you provide permission for this access to your medical records.

Research Participant Consent

I have been verbally informed about the study and I have read the attached written information. I have had the opportunity to ask questions, and these have been answered. The information has included potential benefits and risks of participation.

I understand that participation is entirely voluntary. I am aware that I can withdraw my consent at any time without explanation, and this will not affect my future care.

I confirm that:

- I consent to participate in the study knowing it is voluntary.
- I understand how my personal data will be managed and stored electronically by the researchers.
- I permit access to relevant medical records by the researchers, study monitors, and regulatory authorities for oversight purposes.
- I understand I can withdraw my consent at any time.
- I agree that samples I provide will be stored in a biobank and used for research as described

Results and Reporting

The results will be published in scientific journals and presented at national scientific meetings. Only group-level statistics will be presented, ensuring no individuals can be identified. Information on clinical trials can be found at: www.clinicaltrial.gov.

You may also ask your doctor for updates on study results when available.

Costs, Compensation, and Insurance

As in regular healthcare, you are covered by Patient Injury Insurance.

I hereby give my consent to participate in the study

Signature of research participant Name clarification

______________________________ ____

Date ________________________

I have provided information about the study, and the research participant has had the opportunity to ask questions.

Signature of the clinic personnel

Siganture Name clarification

______________________________ _______________________________

Date___________

A copy of this signed form will be given to the research participant.
