# Supplementary material for: Heart recipient outcomes following transplantation of donor hearts with impaired versus normal function: a study protocol for IMPROVED Heart, a prospective multicentre observational study
Source: BMJ Open. 2026 Jul 10;16(7):e111146. doi: 10.1136/bmjopen-2025-111146 (PMC13358299; doi:10.1136/bmjopen-2025-111146)
Supplement: online supplemental file 2 [file bmjopen-16-7-s002.docx]

List of abbreviations

CPB Cardiopulmonary Bypass

**CRF** Case report form

**DBD** Donation after brain death

**EF** Ejection fraction

**e-CRF** Electronic case report form

**ELISA** Enzyme-linked immunosorbent assay

**GCP** Good Clinical Practice

**GDPR** General Data Protection Regulation

**ICD**  International Statistical Classification of Diseases and Related Health Problems

**ICU**  Intensive care unit

**ISHLT**  International Society for Heart and Lung Transplantation

**LVEF**  Left ventricular ejection fraction

**NTproBNP**  N-terminal pro–B-type natriuretic peptide

PA-catheter Pulmonary artery catheter

**PGD**  Primary graft dysfunction

**PI**  Principal investigator

**REDCap**  Research Electronic Data Capture

**RWMA**  Regional wall motion abnormalities

RRT Renal replacement Therapy

**SAE**  Serious adverse event

**SAP**  Statistical analysis plan

**STRAX**  Swedish Thoracic Transplantation Register

**TTE**  Transthoracic echocardiography
