# Supplementary material for: Heart recipient outcomes following transplantation of donor hearts with impaired versus normal function: a study protocol for IMPROVED Heart, a prospective multicentre observational study
Source: BMJ Open. 2026 Jul 10;16(7):e111146. doi: 10.1136/bmjopen-2025-111146 (PMC13358299; doi:10.1136/bmjopen-2025-111146)
Supplement: online supplemental file 3 [file bmjopen-16-7-s003.pdf]

IMPROVED Heart
List of variables

RECIPIENT DATA

Informed consent

Obtained

Inclusion criterias

all fullfilled

Exclusion criteria

none

Background data

Age

Sex

Body lenght

Body weight

Scandianumber

Medical history

Smoking

Hypertension

Hyperlipidemia

Diabetes, treatment

Pulmonary disease

Pulmonary embolism

Liver disease, type

Kidney disease

Inflammatory disease

Malignancy

Peripheral artery disease

Other

Cardiac disease

Underlying diagnosis leading to tx

Previous heart surgery, type

Previous heart tx

Pacemaker, type

NYHA class

VO2 max (if available)

Echo

Ejection fraction

LVEDD

TAPSE or RV S´

Renal function

Estimated GFR

Measured GFR (if available)

Cardiac catheterization, undilated and dilated

Date
PVR
PCWP
MAP
mPAP
CVP
TPG
CO
CI
SvO2

Cardiac status before tx

- Ongoing hospital care
  - If yes, specify treatments
    - Inotropes
    - Mechanical ventilation
    - Dialysis
    - IABP
    - MCS, type
- Home before tx
  - If yes, specify treatments
    - LVAD
    - BiVAD
    - Other
    - Date for implant
    - None of above
  - Levosimendan within three months
  - Dialysis

Perioperative data

- Date of tx
- Ischemic time
- Perservation device, time on device
- ECC time
- Other simultaneous tx
- Cardioplegia, type

At 24h post-tx (assessment of PGD)

- Hemodynamics
  - CVP
  - PCWP
  - mPAP
  - sPAP
  - Cardiac Output
  - Cardiac Index
  - MAP
- Inotropes
  - Dopamin, y/n and dose

Dobutamin, y/n and dose
Milrinon, y/n and dose
Adrenalin, y/n and dose
Noradrenalin, y/n and dose
Isoprenalin, y/n and dose
Other, type and dose
IABP
MCS
VA-ECMO
LVAD
BiVAD
Perkutan VAD
RVAD
Other
Other reason for heart failure, e.g., acute rejection, pulmonary hypertension

At ICU discharge

Days in ICU
Days with mechanical ventilation
Days with NIV
Days with vasopressor
Days with adrenaline
Days with inotropic support
Days with isoprenaline
Days with NO
Days with flolane
Days with CRRT
Days with MCS and type
VA-ECMO
LVAD
BiVAD
Perkutan VAD
RVAD
Other

At hospital discharge or discharge from transplantation ward

Days on transplantation ward
Discharge to (home, other ward, other hospital, nursing home, dead)
Repeated surgery, type and number
Bleeding
Tamponade
Late closure
Mediastinitis/deep infection
Wound revision
Repeated surgery in groin
Re-tx

Other

Rejection

If yes, specify

Highest grade

Antibodymediated

Serious adverse events

Dead within 28 days

Postoperative MCS need >24 hours post-tx
Other adverse event likely attributable to donor LV dysfunction

Coronary angiography (if available)

At 3, 6 months

Dead, y/n

At one year

Alive or dead, date of death

Re-tx, y/n, date of re-tx

On MCS y/n, date on MCS

Cardiac catheterization, undilated and dilated

Date

PVR

PCWP

MAP

mPAP

CVP

TPG

CO

CI

Echocardiography

Ejection fraction

VTI

Stroke volume

Cardiac index

RWMA, areas with WMA

TAPSE or RV S´

TR Vmax

Renal function tests

Register follow up at one, threee, five and ten years

ICD-codes

Medical procdure codes

Cause of death

DONOR DATA

At identification

- Is the patient a possible donor, are inclusion criteria fulfilled
- Time and date
- Scandia transplant number
- Background
  - Treating hospital
  - Year of birth
  - Sex
  - Body lenght
  - Body weight
  - BMI
  - History of
    - Smoking
    - Hypertension
    - Diabetes, type of treatment
    - Pulmonary disease, type of disease
    - Peripheral arterial disease
    - Hyperlipidemia
    - Kidney disease, type of disease
    - Neurological disease, type of disease
    - Autoimmune disease, type of disease
    - Malignancy, type of malignancy AND active, not active <5 years passed or >5 years passed
    - Drug misuse, type of substance
    - Psyciatric disease, type of disease
    - Suspiction of infection/sepsis
- Clinical data
  - Date of event
  - Start of mechanical ventilation support, date and time
  - Cause of death
    - Cardiac arrest
    - Severe hypoxia
    - Intracerebral haemorrhage
    - Subarachnoid haemorrhage
    - Trauamtic brain injury
    - Ischemic stroke
    - Tumor
    - Other
  - Mechanism
    - Cardiac event
    - Cerebrovascular event
    - Penetrating trauma
    - Blunt trauma
    - Drowning
    - Hanging
    - Intoxication, type of substance
    - Seizures
    - Meningitis
    - Other
  - Clinical determination of brain death through two examinations
    - Date and time
  - Four-vessel cerebral angiography if deemed neccessary, two examinations

        - Date and time
    - Declaration of death
        - Date and time
- Echo (can be repeated)
    - Date and time
    - Hospital
    - LV function
        - Ejection fraction
        - VTI
        - Stroke volume
        - Cardiac index
        - RWMA, areas with WMA
    - RV function
        - TAPSE or RV S'
        - Dilated RV chamber
        - D-sign
    - Pulmonary pressures
        - TR Vmax
        - CVP
    - Valve disorder
        - Type
    - Other
    - Vasoactive drugs at time of echo
        - Noradrenalin, y/n, dose
        - Milrinon, y/n, dose
        - Levosimendan, y/n, dose
        - Dobutamin, y/n, dose
        - Other, type, dose
    - Hemodynamics at time of echo
        - Blood pressure
        - Heart rate
        - Rythm, type
    - Mechanical ventilation, serttings at time of echo
        - PEEP
        - Peak pressure
        - Tidal volym ml/kg/PBW
        - FiO2
    - Lab at time of echoe
        - paO2
        - TNT/TNI (can be repeated)
            - Value
            - Time and date
        - NTproBNP (can be repeated)
            - Value
            - Time and date
    - Metylpredinosolon given?
        - Date, time and dose
    - Neurological examination at time of echo
        - Reaction level scale if not yet declared DBD
        - Pupiles, size and reaction to light if not yet declared DBD

Coronary angiogram
- Normal
- Findings
  - ateromatosis
  - stenosis
  - other

Utilization of donor heart
- Yes
- No
- If no, specify contributing and primary reasons for non-utilization
  - Confirmed heart disease
  - Suspected heart disease
  - Impaired cardiac function
  - Cardiac injury (elevated troponin)
  - Prolonged cardiac arrest
  - Arrhythmias
  - Need for inotropic support
  - Hemodynamically unstable donor
  - No suitable recipient
  - Size mismatch
  - Donor age
  - Donor comorbidity
  - Bloodborne infection
  - Expected prolonged ischaemic time
  - Other donor-related factors
  - Did not progress to brain death
  - Offered to another centre
  - Other
