# Supplementary material for: Heart recipient outcomes following transplantation of donor hearts with impaired versus normal function: a study protocol for IMPROVED Heart, a prospective multicentre observational study
Source: BMJ Open. 2026 Jul 10;16(7):e111146. doi: 10.1136/bmjopen-2025-111146 (PMC13358299; doi:10.1136/bmjopen-2025-111146)
Supplement: online supplemental file 4 [file bmjopen-16-7-s004.pdf]

CLINICAL STUDY PROTOCOL

Title:

IMPact of donor heart function on Recipient Outcomes - a prospectiVE study to increase the utilization of Donor HEARTs

Short title: IMPROVED HEART

Version number:	1.2
Date:	2024-12-08
Sponsor:	Jonatan Oras
Principal Investigator:	Göran Dellgren

Table of contents

Signature page 4
Contact information 5
1. Background and rationale 7
2. Risk-benefit evaluation 9
3. Study objectives 10
3.1. Primary objective 10
3.2. Secondary objectives 10
3.3. Primary variable 11
3.4. Secondary variable 11
4. Study design and procedures 12
4.1. Overall study design 12
4.2. Procedures and flow chart 13
4.3. Biological sampling procedures 16
4.3.1. Handling, storage, and destruction of biological samples 16
4.3.2. Total volume of blood per study subject 16
4.3.3. Biobank 16
4.4. End of study 17
5. Subject selection 17
5.1. Inclusion criteria 17
5.2. Exclusion criteria 17
6. Handling of adverse events 17
6.1. Definitions 17
6.1.1. Adverse Event, AE 17
6.1.2. Serious Adverse Event, SAE 18
6.2. Assessment of adverse events 18
6.2.1. Assessment of causal relationship 18
6.3. Reporting and registration of adverse events 18
6.3.1. Reporting of adverse events (AE) 18
6.3.2. Reporting of serious adverse events (SAE) 18
6.4. Follow-up of adverse events 18
6.5. Independent Data Monitoring Committee 18

7. Statistics 18
7.1. Analysis population 18
7.2. Statistical analyses 19
7.2.1. Statistical methods 19
7.2.2. Drop-outs 19
7.3. Adjustment of significance and confidence interval 19
7.4. Sample size calculations 19
7.5. Interim analysis (if relevant) 19
8. Quality control and quality assurance 20
8.1. Quality assurance and Sponsor oversight 20
8.2. Monitoring 20
8.3. Source data 20
9. Ethics 20
9.1. Compliance to the protocol and regulations 20
9.2. Ethical review of the study 20
9.3. Procedure for obtaining informed consent 21
9.4. Data protection 21
9.5. Insurances 22
10. Substantial changes to the study 22
11. Collection, handling, and archiving data 22
11.1. Case Report Form (Forskningspersonsformulär) 22
12. Notification of study completion, reporting, and publication 23
13. References 23
14. Attachments 25

Signature page

Sponsor

I am responsible for ensuring that this protocol includes all essential information to be able to conduct this study. I will submit the protocol and all other important study-related information to the responsible investigator(s) so that they can conduct the study correctly. I am aware that it is my responsibility to hold the staff members who work with this study informed and trained.

Sponsor's signature	Date
Jonatan Oras	

Printed name

Coordinating Investigator / Principal Investigator

I have read this protocol and agree that it includes all essential information to be able to conduct the study. By signing my name below, I agree to conduct the study in compliance with this protocol, the Declaration of Helsinki, ICH GCP (Good Clinical Practice) guidelines and the national and international regulations governing the conduct of this clinical study.

I will submit this protocol and all other important study-related information to the staff members and responsible investigators who participate in this study, so that they can conduct the study correctly. I am aware of my responsibility to continuously keep the staff members and responsible investigators who work with this study informed and trained.

I am aware that quality control of this study will be performed in the form of monitoring, possibly audit, and possibly inspection.

Coordinating Investigator / Principal Investigator's signature	Date
Göran Dellgren	

Printed name

Contact information

Role in the study	
Sponsor	Jonatan Oras, Senior Specialist, Associate Professor<br>AnOpIVA, Område 5, Sahlgrenska University Hospital<br>Blå stråket 5, vån 5. 413 45 Göteborg<br>+46736370350<br>jonatan.oras@vgregion.se
Principal Investigator	Göran Dellgren<br>Transplantation Centre, Sahlgrenska University Hospital<br>Bruna stråket 5. 413 46 Göteborg<br>+46313428863<br>Goran.dellgren@vgregion.se

Synopsis

Title:	IMPact of donor heart function on Recipient Outcomes - a prospectiVE study to increase the utilization of Donor HEARTs
Short background/Rationale/Purpose:	Severe heart failure is a diagnosis with a very poor prognosis. Heart transplantation is the best treatment for terminal heart failure but this type of treatment is limited by the number of available organs. About 20-25% of possible donor hearts are not considered for transplantation because they have some form of functional impairment. The functional impairment affecting organ donors is, however, usually reversible. A number of retrospective studies show that cardiac function recovers and recipients of hearts with dysfunction do not have worse outcomes compared to recipients of hearts with perfect cardiac function. However, there are no prospective studies on whether the functional impairment of the donor heart is of significance for the recipient. With a systematic and simple investigation of the heart, it should be possible to identify the hearts that are safe to transplant. This will thus increase the number of available donors.
Study objectives:	The objective of the study is to increase the number of available heart donors, which in turn will increase the number of heart transplantations.
Study design:	Prospective, non-randomized multicenter study
Study population:	Patients who are planned to undergo heart transplantation
Number of subjects:	A total of 445 transplanted patients will be included, of which at least 89 patients have received a heart with impaired function.
Inclusion criteria:	Patients who are accepted for heart transplantation at participating centers.<br><br>Aged 18 years or older<br><br>The patient must have provided informed consent
Exclusion criteria:	Mental inability, reluctance or language difficulties that result in difficulty understanding the meaning of study participation.

	Another study, where the patient is included, which is not considered compatible with the current study.
Study outcomes:	Primary variable: Death, retransplantation or need of mechanical assist one year after the heart transplantation.<br><br>Secondary variable: Left-ventricular moderate or severe primary graft dysfunction<br><br>The study has several secondary objectives that are presented in the protocol.
Study period:	Q2 2020 – Q2 2027

1. Background and rationale

Severe heart failure is a diagnosis with a very poor prognosis with a 2-year mortality rate is over 50%, which exceeds many cancer diagnoses[1]. Despite advances in pharmacological treatment and mechanical pumps, heart transplantation is considered to be the best treatment for terminal heart failure[2]. However, heart transplantation as a treatment for terminal heart failure is limited by the number of available organs[3]. If more organs were available, more patients would be transplanted. It is therefore of the utmost importance that every heart that can be successfully transplanted is utilized.

According to current recommendations, a heart should be in near perfect condition to be considered for transplantation[4]. For a donor heart to be transplanted, it should not exhibit:

- More than mild-to-moderate cardiac impairment (EF < 40%)
- Regional wall motion abnormalities
- Need for excessive inotropic support (dobutamine >20µg/kg/minute)
- Intractable ventricular arrhythmias.

However, cardiac dysfunction in organ donors is usually secondary to the disease that led to brain death and is generally reversible, i.e., there is nothing wrong with the heart itself but it has been exposed to an unfavorable environment that temporarily affects its function[5-7]. A common cause of cardiac dysfunction in organ donors is stress-induced cardiomyopathy/Takotsubo cardiomyopathy. Stress-induced cardiomyopathy is a relatively newly described acute cardiac syndrome in which the heart develops regional wall motion abnormalities caused by severe catecholamine stimulation of the myocardium[8-13]. In organ donors, a strong catecholamine storm is observed with brain herniation and the development of brain death, which can trigger stress-induced cardiomyopathy[14,15]. This type of change is seen in 20-25% of potential organ donors[16,17]. One of the most important characteristics of stress-induced cardiomyopathy is its rapid recovery, however, and cardiac function is usually normalized within a few hours or days[8-10,18]. This is not only seen as a functional recovery, but also a structural and biochemical recovery of the heart[10,13]. In addition to organ donors with intracranial events, stress-induced cardiomyopathy is also seen in patients with hypoxic/anoxic brain injuries as a result of hypoxic cardiac arrest, and even in these cases, cardiac events are temporary[19,20].

There are potentially differential diagnoses for stress-induced cardiomyopathy in potential donors. The most important is ischemic heart disease which can quickly and relatively easily be diagnosed with coronary angiography[21]. Coronary angiography is performed today on approximately one-third of potential donors and then primarily on donors with a risk profile for coronary artery disease[22]. Another different diagnosis is myocarditis, which does not have the same reversibility as stress-induced cardiomyopathy and usually have a different echocardiographic pattern.

In the transplant society, it is debated whether a heart with a mild/moderate impaired function can be suitable for transplantation. According to previous recommendations, which have been in place for over 10 years, a heart must be in nearly perfect condition to be considered for transplantation[4]. These guidelines lacked scientific evidence but have gained significant acceptance, leading to caution in transplanting such hearts [23]. In a survey of American thoracic surgeons, reduced ejection fraction and regional hypokinesia were ranked as the most important factors for not accepting a heart. Furthermore, these were also identified as the primary reasons for rejecting a heart for transplantation[17].

There is a number of retrospective studies that show that the outcome is not worse for patients who have been transplanted with hears that have/have had dysfunction[16,24,25]. The main studies are presented below:

- A study based on material from the USA (UNOS database) in which patients who received a heart with ejection fraction <50% (n=740) were identified and compared with recipients of hearts with normal function (n=30253). No difference in mortality at one year after transplantation was observed between the groups[25].
- A study based on the same material showed that long-term survival was not inferior for recipients of hearts with ejection fraction<50%. Cardiac function was also the same for recipients of hearts with normal function and impaired function one year after transplantation[26].
- In a study based on the same database (UNOS database) 472 recipients who received a heart with improved function, defined as EF≤40% at one examination and EF≥50% at a follow-up examination, were identified. The control group were recipients of hearts with normal function EF≥55%. There was no difference in mortality, primary graft failure, allograft vasculopathy between the groups, also when propensity score matching was applied[24].
- A study based on other material from the California Transplant Donor Network reported data where low ejection fraction (EF<50%, number of patients not reported) or regional hypokinesia (n=197) did not affect outcome[16].
- An older study based on material from the USA showed that regional hypokinesia has no bearing on recipients' mortality. The number of patients with regional hypokinesia was not reported but 1719 patients were included in the study[27].
- A study from Transplantation Centre, Sahlgrenska, analyzed donors and recipients during a 10-year period. In total, 641 donors were included in the analyses and 155 (24%) of the donors had cardiac dysfunction. Recipients of hearts with dysfunction (n=42) did not have a higher risk of adverse outcomes (death, retransplantation) compared to recipients of hearts with normal function. Short-term outcomes (intensive care unit (ICU) care time, advanced hemodynamic support, postoperative dialysis, rejection) did not differ between the groups. Cardiac function was rapidly recovered in the recipients and both groups had the same ejection fraction already a few days after transplantation[28].

These studies are the foundation for recently published guidelines regarding which hearts can be considered for transplantation[29] . They suggest that hearts with initially impaired function should be further evaluated, and those that demonstrate functional improvement could be considered for transplantation. However, the level of evidence is low, and the strength of recommendation is moderate (Level of Evidence: C, Level of Recommendation II). There is a lack of prospective studies on the transplantation of hearts with impaired function, which contributes to the low level of evidence and the fact that clinical practice has not changed

In Sweden, there are many years of experience of handling hearts with impaired function. This clinical experience suggests that it is not associated with an increased risk of complications. The study referred to above confirm this experience[28].

In summary, there is a potential to increase the number of heart donors, and thus the number of heart transplantations, if the heart donors with reversible dysfunction are utilized. This study aims to prospectively investigate the cardiac function of donors and evaluate if it is of significance to the outcome of the recipient. The study sets guidelines for investigative procedures as well as which hearts should be transplanted, based on previous retrospective studies. We expect that the recipients' outcome will not be affected by the cardiac function of the donor, provided the study's recommendations are followed. Our data shows that approximately every fourth potential heart donor suffers from functional impairment. This is in line with other studies that observed that about 20% of hearts are refused due to affected function[17]. We estimate that a systematic utilization of these hearts can increase the number of transplantations by 20-30%.

2. Risk-benefit evaluation

Today, patients are transplanted with donor hearts with impaired function. In the vast majority of cases, such transplantations occur according to the recommendations given in this study (section 5.1). In retrospective data, these patients do not have worse outcomes in the short- or long-term, also in adjusted data. However, in retrospective studies we do not have control of all covariates and there may be factors that the responsible surgeon took into account which could not be measured. Thus, there is a conceivable risk that patients have an increased risk for complications, if the utilization of such hearts would be more liberal.

The overall benefit of utilizing donor hearts with impaired function is the recipient's shorter time waiting for an organ. For the recipient, this means a shorter time on the waiting list. While waiting for transplantation it is not uncommon that cardiac function deteriorates. The patient might be in need of mechanical assist and in worst case, the patient dies. In addition to the reduction of these risks, the patient's time with a disabling disease is decreased.

The study will continuously evaluate the results to find an increased risks for the group who has received a heart with impaired function. In such cases, the study will be prematurely terminated.

From a larger perspective, more available heart donors will allow patients to be more liberally accepted for transplantation. In the long-term, we hope that this can change the guidelines for which patients are relevant for transplantation. Our hope is also that fewer patients will need mechanical assist, a treatment that is expensive and limits daily activities.

This study will not expose heart recipients to any examinations or examinations other than those performed as part of clinical follow-up, which means that there is no additional suffering for the patient in the study follow-up.

Based on ours and others' retrospective data, there is no increased risk from receiving a heart that has impaired function. The guidelines set up in this study are based on studies of 1400 reported patients. Based on the state of knowledge today, we expect that these heart recipients will not have an increased risk for complications or death. The benefit for the patients is that they will receive their transplantation faster, which is a favorable factor. Taken together, we estimate that the potential benefit outweighs possible risks.

3. Study objectives

The aim of this study is to increase the number of heart transplantations by increasing the number of available donor heart. We will do this by showing that it is safe to transplant hearts with impaired function, provided that they are investigated and that the functional impairment is within the scope of the study guidelines. The study also aims to facilitate and speed up the investigation of possible heart donors. We will also learn more about cardiac dysfunction in organ donors through access to tissue samples.

We estimate that the number of heart transplantations can increase by 20-30% if these donors can be systematically utilized. In practice, this means that the transplantation queue can be reduced and that patients can more liberally accepted for heart transplantation.

3.1. Primary objective

The primary objective is to investigate whether recipients of donor hearts with regional wall motion abnormalities and/or mild to moderate globally reduced cardiac function have the same outcome as recipients of hearts with normal function.

3.2. Secondary objectives

The secondary research questions that this study intends to answer are the following:

Does cardiac function differ postoperatively in a recipient who has received a donor heart with a functional impairment compared to a recipient who has received a donor heart with normal function?

With a systematic consideration of hearts with impaired function, by how much do the number of heart transplantations increase and how much faster can a patient be transplanted?

Does cardiac function in the donor affect cardiovascular events over time in the heart-transplanted patients?

Using cardiac biomarkers, ECG, echocardiographic imaging, and clinical data, can it be predicted early on which hearts will improve/recover function during the donation process?

Using cardiac biomarkers, ECG, echocardiographic imaging, and clinical data, can cardiac dysfunction caused by primary heart disease be ruled out?

Do donor hearts with dysfunction have different histology and/or biochemistry?

3.3. Primary variable

The primary variable is: Death, retransplantation or need of a mechanical assist one year after heart transplantation.

3.4. Secondary variable

The secondary variable is: frequency of left-sided moderate or severe primary graft dysfunction (PGD)[30].

Moderate PGD is defined as having *at least one of the following at 24 hours after cross-clamp release:*

- Ejection fraction ≤ 40%, *or* hemodynamic compromise with right atrial pressure (RAP) > 15mmHg, pulmonary capillary wedge pressure > 20mmHg, cardiac index < 2.0, hypotension with a mean arterial pressure < 70mmHg
  And at least one of the following:
- High-dose inotropes (Inotrope score >10) *or* newly placed intra-aortic balloon pump.

Severe PGD is defined as dependence on left or biventricular mechanical support in the form of either ECMO, LVAD, BiVAD, or percutaneous LVAD at 24 hours after cross-clamp release.

The following outcome variables apply to the secondary research questions:

Need for interventions and complications in the postoperative course including, for example, mechanical heart pump, intra-aortic balloon pump, need for mechanical ventilation, need for dialysis, dose and time with inotropic and vasoactive medications.

Occurrence of ICD codes for cardiovascular disease in the Patient and Cause of Death registries for participating patients at 3, 5, and 10 years after transplantation.

Levels of cardiac biomarkers, occurrence of specific echocardiographic changes, ECG changes, and clinical data in donors with recovered and non-recovered cardiac function during the donation process, respectively.

Levels of cardiac biomarkers, occurrence of specific echocardiographic changes, ECG changes, and clinical data in donors with primary and non-primary heart disease, respectively.

Estimation of the number of extra patients who would be able to receive a heart transplantation with a systematic utilization of donor hearts with dysfunction.

Presence of specific histological changes and specific proteins in heart tissue with dysfunction and normal function, respectively.

4. Study design and procedures

4.1. Overall study design

This is a non-randomized study.

The study consists of two protocols, one for the possible donors and one for the heart recipients. The heart function of the donor is systematically investigated according to the assessment protocol. The heart recipient is followed up according to clinical routine. Participation in the study will not affect what organ is allocated to the recipient. This procedure is according to clinical routine. The respective sections are presented below.

Assessment of donor hearts (Attachment 1)

For a possible donor to be relevant to the study, they must meet the following criteria. (1) The person shall be identified as a possible donor, (2) next of kin should have been informed about futile prognosis and transition to end-of-life care, (3) the person shall fulfill criteria(s) for being positive to organ donation according to national guidelines / local regulations and (4) the transplantation coordinator / center have been contacted. For a possible donor where all criteria are met, an ultrasound examination of the heart is planned as soon as it is suitable and logistically possible. In cases where the donation process proceeds, cardiac examination follows the protocol (Figure 1). This can briefly be described in three different scenarios.

- Scenario 1: If the UCG shows normal cardiac function, the heart is accepted for transplantation in the usual way.
- Scenario 2: If the UCG shows ejection fraction < 50%, an assessment of whether the cardiac function is acceptable according to set recommendations (see below) is performed. If the function is not considered acceptable, echocardiography is repeated when practically feasible. An improvement in ejection fraction could be expected after 12 hours. This can be repeated several times, until the donation process ends.
- Scenario 3: If the UCG shows regional hypokinesia and coronary artery disease cannot be ruled out clinically, a coronary angiogram or CT-heart is performed. If the coronary angiogram excludes coronary artery disease, assessment of the cardiac function is done according to scenario 2.
-

At each UCG, investigations with ECG and blood tests for cardiac biomarkers (troponin T, NTproBNP) are performed. Clinical data will be recorded from the actual care event and during the donation process.

Recommendations for accepting marginal hearts:

- Ejection fraction shall be over 40%
- Improvement (but not necessarily normalization) of cardiac function should be observed. The study recommends an improvement in ejection fraction of 10% to be significant.

- Regional wall motion abnormalities are not in themselves a contraindication for heart transplantation, unless coronary artery disease is excluded according to clinical guidelines or with coronary angiogram.
- In cases with borderline ejection fraction (slightly below 40%), inotropic support may be given to evaluate the reversibility of cardiac function (test with inotropes).
- Cardiac biomarkers with relatively low troponin values that are decreasing, and high NTproBNP is supportive for a decision to transplant the heart.

These recommendations are based on the retrospective studies available[16,18,24,25]. The protocol ensures that the cardiac dysfunction observed is a dynamic and reversible process. The recommendation of having an increase in ejection fraction of 10% to be considered significant reduces the risk of misassesments. The decision for transplantation of the donor heart remains with the responsible thoracic surgeon, as there may be additional factors that the study protocol cannot take into account. In such cases, these will be documented.

Follow-up of recipients

Recipient baseline data, including demographics and medical history, is recorded at inclusion. The recipients will be followed regarding death, need of mechanical heart pump and re-transplantation during the first year (primary outcome variable). Short-term data will be collected regarding need of post-operative mechanical assistance, intra-aortic balloon pump, dialysis, time with mechanical ventilation, intropic drugs, re-surgery(s), time at the ICU, time at the home clinic/other department, time before returning home. The recipients will be followed according to clinical routine at the participating center, and no extra examinations are planned, with the exception that echocardiography is preferable performed at 24 ± 6 hours for assessment of PGD.

The active follow-up end at one year after transplantation. Long-term complications that will be followed are death, need for mechanical heart pump, re-transplantation, rejection/coronary vasculitis, other organ failure. To investigate whether recipients of hearts with dysfunction have increased risk for other disease, data will be collected from the Swedish National Board of Health and Welfare's (Socialstyrelsen) Patient Register and Cause of Death Register. Those data that will be requested are all of the patients' ICD codes and medical procedure codes at 1, 3, 5, and 10 years after transplantation. Data may be collected from other national registers if they are available.

A list of variables is available in *Attachment 2.*

4.2. Procedures and flow chart

Follow-up is done according to clinical routine at each participating center with the exception that echocardiography is preferable performed at 24 ± 6 hours for assessment of PGD. A study visit scheme is available in *Attachment 3.*

Data will be collected from the donor during the donation process. Data from recipients will be collected at the following time points: at inclusion, at the time of transplantation, 24 hours post-transplantation, at discharge from the intensive care unit (can be repeated upon readmission), at

discharge from the hospital, SAE monitoring up to 28 days, mortality follow-up at 3 and 6 months, and at one year post-transplantation.

Instructions to register data follows below:

Pre-op Data / Transplantation

- The data recorded is the data that forms the basis for the decision to transplantat. This is particularly important for echocardiography, cardiac catheterization, VO2 max, and GFR, where multiple examinations may exist. Typically, the examinations closest to the transplantation decision are used.
- If the patient is accepted for mechanical assist as a bridge-to-transplant, the data underlying the bridge-to-transplant decision is registered.
- For patients receiving mechanical assist prior to the initiation of the transplantation evaluation (e.g., in cases of acute need for mechanical assist, i.e., "bridge-to-decision"), echocardiography, cardiac catheterization data, and GFR/eGFR before mechanical assist are registered, if available. If not available, the data in closest proximity to mechanical assist is registered.
- For GUCH (Grown-Up Congenital Heart) patients, the available data is recorded. The ejection fraction for the systemic ventricle is reported.

At Transplantation

- The data registered is that which is available closest to the time of transplantation. For echocardiography, cardiac catheterization, VO2 max, and GFR, the data closest to transplantation is registered. If no new data has been collected since acceptance for transplantation, no new data needs to be registered.
- For patients with mechanical assist, echocardiography data is not registered. Cardiac catheterization data is registered at the baseline speed setting. Register the data available, noting that dilated values are typically not present. Pressure values are more important than cardiac output (PVR, PCWP, mPAP, CVP, TPG). GFR/eGFR is registered if available.
- Ischemic time is defined as the time from flushing to "cross-clamp release." For hearts that have used a heart box, both total ischemia time and time in the box are registered.
- Cross-clamp release marks the time of transplantation. The date and time are recorded from this point.

24 Hours After Transplantation

- If data is missing at the 24-hour mark, data is registered as close as possible to 24 hours, with the following exceptions:
  - Data from a pulmonary artery catheter is valid within ± 6 hours of the 24-hour mark. Values outside this range are considered "missing."

- The first echocardiography is registered in the eCRF under a separate heading. The date and time of the examination are recorded.

At Discharge from ICU

- For ICU interventions (e.g., time with mechanical ventilation, CRRT etc.), any day with an intervention, regardless of how brief it is, is counted as a full day of treatment.
- The ICU discharge form is repeated upon readmission to the ICU.
- Register the echocardiography data as close as possible to discharge from the ICU. If the echocardiography performed at ICU discharge is the same as that performed at 7–14 days, it does not need to be registered again.

Echocardiography at 7–14 Days Post-Transplantation

- The acceptable range for echocardiography is 7 to 14 days post-transplantation. If no echocardiography is performed during this period, the value is recorded as "missing."
- If multiple echocardiography results are available, register the one closest to 7 days post-transplantation.
- Basic echocardiography, such as screenings for pericardial effusion, does not need to be registered. If only such echocardiography is available within this period, register the data that is available; typically, ejection fraction or heart function is reported.

Discharge from the Transplantation Center

- Register the number of re-operations using the data available in the CRF. Procedure codes from the surgical report should be used.
- For VAC treatment, register the duration of time with VAC.
- Register the echocardiography closest to the time of discharge.
- Days at the transplantation center are counted as the number of days from transplantation to discharge.

SAE Monitoring

- Serious Adverse Events (SAEs) are monitored during the first 28 days post-transplantation. SAEs can be repeated if multiple events occur in the same patient.

Mortality Follow-Up

- Mortality follow-up is conducted at 3 and 6 months post-transplantation.

At 1 Year

- Register the data available closest to 1 year post-transplantation.
- Primarily register data from examinations performed at the respective transplantation center.

Study visit schedule

	Inclusion	Transplantation	24 h after tx	7-14 days after tx	ICU discharge	Discharge from tx-ward	3, 6 months after tx	1 year after tx
Informed consent	x							
Background	x							
Medical history	x							
Cardiac status at inclusion	x							
Echocardiogrpahy	x*	x*	x	x*	x*	x*		x*
Renal function	x*	x*						x*
Cardiac catetherization	x*	x*						x*
Cardiac status at tx		x						
Perioperative data		x						
Basic donor data		x						
Hemodynamic data			x					
Days with MCS					x			
ICU interventions					x			
Repeated surgery						x		
Rejection						x		x
Events and time on ward						x		
Mortality check							x	
Coronary angiography								x
SAE			Registered first 28 days after tx					

Tx; transplantation, inkl; inclusion, PGD; primary graft dysfunction, MCS; mechanical circulatory support, ICU; intensive care unit, SAE; serious adverse events
* if data is available

4.3. Biological sampling procedures

No additional samples will be taken from the research subjects (e.g. the heart recipients).

In cases where it is logistically possible and permission has been granted, in accordance with current regulations, blood and heart tissue will be obtained from the organ donors for histological and biochemical analyses.

4.3.1. Handling, storage, and destruction of biological samples

The hearts of organ donors that are not transplanted may be used for tissue analysis, when logistically feasible and permission is granted, in accordance with national regulations. The hearts will be examined for coronary heart disease and myocardial infarction. Tissue samples will be taken from several areas with affected (in such donors) and normal function. Histological analyses, Western blot, and analyses for proteomics are planned. The analyses are planned to be performed at Wallenberg lab as well as the Core facilities at Sahlgrenska Academy.

Blood samples from donors will be taken when logistically feasible and permission is granted, in accordance with current regulations. Blood samples are planned to be analyzed by enzyme-linked immunosorbent assay (ELISA). The analyses are planned to be performed at Wallenberg lab and Lab of immunology, Sahlgrenska Academy.

Samples will be saved up to 10 years after the study has ended.

4.3.2. Total volume of blood per study subject

No blood will be taken from the recipients.

From the donors, 20 ml blood is planned to be taken on up to three occasions, i.e. a total of 60 ml blood. Blood samples for analysis of troponin T and NTproBNP will be taken as part of normal healthcare routines and destroyed immediately after analysis.

4.3.3. Biobank

All samples taken in this study are registered in a biobank at Biobank West and handled according to current biobank laws and regulations. .The law regulates the way in which samples can be stored and used as well as rules on quality and security for biobanks. The samples are coded/pseudonymized to protect the research subjects’ identification. All samples and the identification list/code list are stored securely and separately to prevent unauthorized person from having access to them.

4.4. End of study

The study ends when the following two criteria are met. (1) 445 patients heart-transplanted patients are included of which (2) at least 89 patients have received a heart with impaired function. The study will continue until both criteria are met.

The study may be prematurely terminated if it appears that the treatment involves a large number of undesirable severe adverse events or if recruitment of research subjects cannot be met within reasonable time limits.

Decisions on premature termination are taken by the sponsor.

5. Subject selection

5.1. Inclusion criteria

To be included in the study, research subjects must meet the following criteria:

- Patient who has been accepted for heart transplantation at the participating transplantation center
- The research subject has given written consent to participate in the study
- Aged 18 years or older

We refer to research subjects only as those who are heart recipients in this study; the heart donors are not considered research subjects since they are deceased.

5.2. Exclusion criteria

The study has the following exclusion criteria:

- Mental inability, reluctance or language difficulties that result in difficulty understanding the meaning of study participation
- Another study, where the patient is included, which is not considered compatible with the current study.

6. Handling of adverse events

6.1. Definitions

6.1.1. Adverse Event, AE

After heart transplantation, a patient usually requires intensive care with circulatory support, respiratory care, and often dialysis. Such treatment is part of the usual course following heart transplantation and will therefore be difficult to assess as unwanted adverse events. We have therefore not defined any adverse events.

6.1.2. Serious Adverse Event, SAE

Serious unwanted events include conditions that require more invasive measures or death. SAE include:

- Need of postoperative mechanical assistance more than 24 hours post-op
- Death within 28 days from transplantation
- Other event that resulted in prolonged care time or suffering for the patient (e.g., stroke with sequelae) that can be attributed to, or is suspected to be attributable to, cardiac function of the donor

6.2. Assessment of adverse events

6.2.1. Assessment of causal relationship

We believe that AE and SAE will be difficult to attribute to cardiac function of the organ donor. Each SAE in particular will be analyzed in the case there may be a causal relationship with the cardiac function of the organ donor. The incidence of AE and SAE between groups will be analyzed in interim analyses.

6.3. Reporting and registration of adverse events

6.3.1. Reporting of adverse events (AE)

AE will not be reported (see above).

6.3.2. Reporting of serious adverse events (SAE)

Serious adverse events (SAE) will be reported to the sponsor on a special SAE form within 24 hours of the investigator being informed of the SAE.

Follow-up information describing the outcome and handling of the SAE is reported as soon as this information is available. The original should be kept in the Investigator Site File.

6.4. Follow-up of adverse events

In case of increased frequency of SAE at the interim analysis, the study may be discontinued.

6.5. Independent Data Monitoring Committee

Since the study is not blinded or randomized, no independent safety committee is planned.

7. Statistics

7.1. Analysis population

The primary variable will be calculated for those patients who are transplanted (per protocol). For some secondary variables (time to transplantation, estimation of the number of increased transplantations), people who were not transplanted will also be included in the analysis (intention to treat).

7.2. Statistical analyses

7.2.1. Statistical methods

The primary variables will be analyzed with binary logistic regression. The primary outcome variable will be incidence of death, re-transplantation or need of long-term mechanical assistance within one year after transplantation. The primary explanatory variable is incidence of cardiac dysfunction in the donor, defined as regional hypokinesia and/or ejection fraction<50%. The analysis will be adjusted for age of recipient, age of donor, need of mechanical assistance before transplantation and "urgent call" status of transplantation.

For statistical significance, the p-value must be >0.05.

7.2.2. Drop-outs

If the primary outcome variable and the primary explanatory variable are missing, the patient will be censored from the analysis. Adjusting variables will be imputed with regression-based imputation.

7.3. Adjustment of significance and confidence interval

In cases where multiple significance (multiplicity) can occur, data will be adjusted with Bonferroni Holm.

7.4. Sample size calculations

This is a non-inferiority trial. Power calculations are made based on non-inferiority calculations. Based on our retrospective data, the absence of death/retransplantation within a year was 90%. We have calculated power to exclude a difference in the primary outcome of 10%, which is the benefit that may be relevant since patients can be transplanted earlier and more patients can be transplanted. We expect that 20% of the transplantations will come from hearts with dysfunction. The power calculation says then that we need 445 transplanted hearts and at least 89 transplanted hearts with dysfunction. Since we do not know the exact incidence, outcome, or transplantation frequency of hearts with dysfunction in a prospective cohort, we primarily plan to include 500 heart-transplanted patients in the study.

To be able to answer our specific research questions about the heart donors, we estimate that we need at least 100 donors of hearts with dysfunction to be able to have a representative group. With an estimated incidence of cardiac dysfunction in 25% of the donors, at least 400 possible heart donors are needed in the study. However, the study intends to continue collecting data from all donors until the primary objective is answered.

7.5. Interim analysis (if relevant)

Interim analysis will be performed after 100 patients have been analyzed for the primary outcome and then after every 50 patients who have been analyzed for the

primary outcome. Criteria for stopping the study are a confirmed overrepresentation of primary outcome variables in the group of patients who received a heart from a donor with impaired cardiac function. Overrepresentation must be at least 10 absolute percent in the primary outcome measure as well as show statistical significance ($p<0.05$) for the study to be terminated. An increased incidence of SAE of at least 20 absolute percent as well as statistical significance ($p<0.05$) can result in termination of the study.

8. Quality control and quality assurance

8.1. Quality assurance and Sponsor oversight

The sponsor will ensure that the study personnel are trained and updated about the study. Meetings (local, regional, and central) will be held to provide updates about study progress and to enable review of any potential problems. Study personnel will be trained and updated through meetings and updating of documents. The study will be monitored.

8.2. Monitoring

To ensure that the study is conducted according to the protocol and that data is collected, documented, and reported in accordance with good clinical practice and applicable ethical requirements, the study will be monitored by an independent monitor before the study begins, during the study conduct, and after the study has been completed. Monitoring is performed according to the study's monitoring plan and is intended to ensure that the research subjects' rights, safety, and well-being are respected as well as that the data in the CRF are complete, correct, and consistent with source data.

8.3. Source data

The investigator must keep source documents for each research subject in the study. A document describing what has been classified as source data as well as which source data is specified in the CRF. The investigator must ensure that all source documents are accessible for monitoring and other quality control activities.

Source data is defined before study start at each individual site (prövningsställe).

9. Ethics

9.1. Compliance to the protocol and regulations

The study will be performed in accordance with the study protocol, the latest version of the Declaration of Helsinki and applicable regulatory requirements. This is to ensure the safety and integrity of the research subjects as well as the quality of the collected data.

9.2. Ethical review of the study

The final study protocol, including the final versions of the informed consent form and other information provided to research subjects, must first be approved or given a written positive

opinion by the Swedish Ethical Review Authority (Etikprövningsmyndigheten). The Swedish Ethical Review Authority must be informed of any changes in the study protocol in accordance with applicable requirements.

9.3. Procedure for obtaining informed consent

Heart recipients will be asked about inclusion in the study during the time window from when he/she is accepted for transplantation until he/she is transplanted. In the first place, inclusion will be requested in connection with the visit in which the patient is accepted for transplantation. The physician who is responsible for the transplant investigation or study personnel, well initiated in the study, will ask about inclusion. Oral and written information will be provided. The patient will also be given the opportunity to ask questions. The patient can go home and provide a response about study participation later. Consent is documented in writing.

The principal investigator at each site shall ensure that the subject is given full and adequate oral and written information about the study, its purpose, any risks and benefits as well as inclusion and exclusion criteria. Research subjects must also be informed that they are free to discontinue their participation in the study at any time without having to provide a reason. Research subjects should be given the opportunity to ask questions and be allowed time to consider the provided information. If the person chooses to participate, both the research subject and the investigator shall sign the informed consent form. A copy of the subject information form as well as the informed consent form shall be provided to the research subject. The research subject’s signed and dated informed consent must be obtained before performing any study-specific activity in the study. Each research subject who participates in the study will be identified by a subject number on a subject identification list. The research subject agrees that monitors and inspectors may have access to their medical records. If new information is added to the study, the research subject has the right to reconsider whether he/she will continue their participation.

9.4. Data protection

If any part of the data is handled by any other organization, inside or outside the European Union, appropriate agreements and/or other documentation will be established, to ensure that the data processing is performed in accordance with the provisions of the General Data Protection Regulation and other relevant legislation, before any data transfer takes place.

The content of the informed consent form complies with relevant integrity and data protection legislation. In the subject information and the informed consent form, the research subject will be given complete information about how collection, use and publication of their study data will take place. The subject information and the informed consent form will explain how study data are stored to maintain confidentiality in accordance with national data legislation. All information processed by the sponsor will be pseudonymized and identified with <<Study code/Study ID/Initials>>.

The informed consent form will also explain that for verification of the data, authorized representatives of the sponsor, as well as relevant authorities, may require access to parts of

medical or study records that are relevant to the study, including the research subject's medical history.

9.5. Insurances

Research subjects are insured by Swedish Patient Insurance (*patientskadeförsäkringen*) during the study.

10. Substantial changes to the study

Substantial changes to the signed study protocol are only possible through approved protocol amendments and by agreement from all responsible persons. Information on non-substantial changes will be clearly noted in the amended protocol.

In the event that substantial changes to the protocol (e.g., changing of the primary objective, primary or secondary variables, method to measure the primary variable, changing of the investigational product or dosage) will be made during the course of the study, approval from the Swedish Ethical Review Authority (EPM) shall be obtained before any changes are implemented. A change that concerns a new site, new investigator or a new study patient information sheet shall also be approved by EPM.

Non-substantial changes will be recorded and later entered in documentation that is submitted, for example in any subsequent notifications of a substantial change or in connection with End of Trial reporting.

11. Collection, handling, and archiving data

Research subjects who participate in the study are coded with a specific study identification number. All research subjects are registered in a subject identification list (subject enrolment and identification list) that connects the research subject's name and personal number with a study identification number.

All data will be registered, managed, and stored in a manner that enables correct reporting, interpretation, and verification. Source documents will be archived for at least 10 years after the study is completed. Source data in the patient records system is stored and archived in accordance with the respective local regulations.

11.1. Case Report Form (Forskningspersonsformulär)

A Case Report Form (CRF) is used for data collection. The study uses an electronic CRF (eCRF). The investigator shall ensure that data is registered and any corrections in the CRF are made as stated in the study protocol and in accordance with instructions. The investigator shall ensure that the registered data is correct, complete, and that reporting

takes place according to the timelines that have been predefined. The investigator signs the completed CRF. A copy of the completed CRF will be archived at the study site.

12. Notification of study completion, reporting, and publication

The primary objective is planned to be published within one year from the study's completion. Data regarding the secondary objectives (regarding the donors as well as tissue analyses) may be published before the primary objective is addressed and published.

13. References

1. Shah KS, Xu H, Matsouaka RA, et al. Heart Failure With Preserved, Borderline, and Reduced Ejection Fraction: 5-Year Outcomes. Journal of the American College of Cardiology 2017;70:2476-86.
2. Ponikowski P, Voors AA, Anker SD, et al. 2016 ESC Guidelines for the diagnosis and treatment of acute and chronic heart failure: The Task Force for the diagnosis and treatment of acute and chronic heart failure of the European Society of Cardiology (ESC)Developed with the special contribution of the Heart Failure Association (HFA) of the ESC. European heart journal 2016;37:2129-200.
3. Branger P, Samuel U. Eurotransplant international foundation annual report 2016. CIP-Gegevens Koniklijke bibliotheek, Den Haag 2017.
4. Costanzo MR, Dipchand A, Starling R, et al. The International Society of Heart and Lung Transplantation Guidelines for the care of heart transplant recipients. The Journal of heart and lung transplantation : the official publication of the International Society for Heart Transplantation 2010;29:914-56.
5. Dujardin KS, McCully RB, Wijdicks EF, et al. Myocardial dysfunction associated with brain death: clinical, echocardiographic, and pathologic features. The Journal of heart and lung transplantation : the official publication of the International Society for Heart Transplantation 2001;20:350-7.
6. Mohamedali B, Bhat G, Zelinger A. Frequency and pattern of left ventricular dysfunction in potential heart donors: implications regarding use of dysfunctional hearts for successful transplantation. Journal of the American College of Cardiology 2012;60:235-6.
7. Zaroff JG, Babcock WD, Shiboski SC, Solinger LL, Rosengard BR. Temporal changes in left ventricular systolic function in heart donors: results of serial echocardiography. The Journal of heart and lung transplantation : the official publication of the International Society for Heart Transplantation 2003;22:383-8.
8. Wittstein IS, Thiemann DR, Lima JA, et al. Neurohumoral features of myocardial stunning due to sudden emotional stress. The New England journal of medicine 2005;352:539-48.
9. Templin C, Ghadri JR, Diekmann J, et al. Clinical Features and Outcomes of Takotsubo (Stress) Cardiomyopathy. The New England journal of medicine 2015;373:929-38.
10. Ghadri JR, Wittstein IS, Prasad A, et al. International Expert Consensus Document on Takotsubo Syndrome (Part I): Clinical Characteristics, Diagnostic Criteria, and Pathophysiology. European heart journal 2018;39:2032-46.
11. Paur H, Wright PT, Sikkel MB, et al. High levels of circulating epinephrine trigger apical cardiodepression in a beta2-adrenergic receptor/Gi-dependent manner: a new model of Takotsubo cardiomyopathy. Circulation 2012;126:697-706.

12. Shao Y, Redfors B, Scharin Tang M, et al. Novel rat model reveals important roles of beta-adrenoreceptors in stress-induced cardiomyopathy. Int J Cardiol 2013;168:1943-50.
13. Akashi YJ, Nef HM, Lyon AR. Epidemiology and pathophysiology of Takotsubo syndrome. Nature Reviews Cardiology 2015;12:387-97.
14. Mertes PM, Carteaux JP, Jaboin Y, et al. Estimation of myocardial interstitial norepinephrine release after brain death using cardiac microdialysis. Transplantation 1994;57:371-7.
15. Chiari P, Hadour G, Michel P, et al. Biphasic response after brain death induction: prominent part of catecholamines release in this phenomenon. J Heart Lung Transplant 2000;19:675-82.
16. Khush KK, Menza R, Nguyen J, Zaroff JG, Goldstein BA. Donor predictors of allograft use and recipient outcomes after heart transplantation. Circulation Heart failure 2013;6:300-9.
17. Tryon D, Hasaniya NW, Jabo B, Razzouk AJ, Bailey LL, Rabkin DG. Effect of left ventricular dysfunction on utilization of donor hearts. The Journal of heart and lung transplantation : the official publication of the International Society for Heart Transplantation 2018;37:349-57.
18. Oras J, Grivans C, Dalla K, et al. High-Sensitive Troponin T and N-Terminal Pro B-Type Natriuretic Peptide for Early Detection of Stress-Induced Cardiomyopathy in Patients with Subarachnoid Hemorrhage. Neurocritical care 2015.
19. Oras J, Lundgren J, Redfors B, et al. Takotsubo syndrome in hemodynamically unstable patients admitted to the intensive care unit - a retrospective study. Acta anaesthesiologica Scandinavica 2017;61:914-24.
20. Cha KC, Kim HI, Kim OH, et al. Echocardiographic patterns of postresuscitation myocardial dysfunction. Resuscitation 2018;124:90-5.
21. Roffi M, Patrono C, Collet JP, et al. 2015 ESC Guidelines for the management of acute coronary syndromes in patients presenting without persistent ST-segment elevation: Task Force for the Management of Acute Coronary Syndromes in Patients Presenting without Persistent ST-Segment Elevation of the European Society of Cardiology (ESC). European heart journal 2015.
22. Zaroff JG, Rosengard BR, Armstrong WF, et al. Consensus conference report: maximizing use of organs recovered from the cadaver donor: cardiac recommendations, March 28-29, 2001, Crystal City, Va. Circulation 2002;106:836-41.
23. Kobashigawa J, Khush K, Colvin M, et al. Report From the American Society of Transplantation Conference on Donor Heart Selection in Adult Cardiac Transplantation in the United States. American Journal of Transplantation 2017;17:2559-66.
24. Madan S, Saeed O, Vlismas P, et al. Outcomes After Transplantation of Donor Hearts With Improving Left Ventricular Systolic Dysfunction. Journal of the American College of Cardiology 2017;70:1248-58.
25. Chen CW, Sprys MH, Gaffey AC, et al. Low ejection fraction in donor hearts is not directly associated with increased recipient mortality. The Journal of heart and lung transplantation : the official publication of the International Society for Heart Transplantation 2017;36:611-5.
26. Sibona A, Khush KK, Oyoyo UE, et al. Long-term transplant outcomes of donor hearts with left ventricular dysfunction. The Journal of thoracic and cardiovascular surgery 2019;157:1865-75.
27. Young JB, Naftel DC, Bourge RC, et al. Matching the heart donor and heart transplant recipient. Clues for successful expansion of the donor pool: a multivariable, multiinstitutional report. The Cardiac Transplant Research Database Group. The Journal of heart and lung transplantation : the official publication of the International Society for Heart Transplantation 1994;13:353-64; discussion 64-5.
28. Oras J, Doueh R, Norberg E, Redfors B, Omerovic E, Dellgren G. Left ventricular dysfunction in potential heart donors and its influence on recipient outcomes. The Journal of thoracic and cardiovascular surgery 2019.

29. Copeland H, Knezevic I, Baran DA, et al. Donor heart selection: Evidence-based guidelines for providers. The Journal of heart and lung transplantation : the official publication of the International Society for Heart Transplantation 2023;42:7-29.
30. Kobashigawa J, Zuckermann A, Macdonald P, et al. Report from a consensus conference on primary graft dysfunction after cardiac transplantation. The Journal of heart and lung transplantation : the official publication of the International Society for Heart Transplantation 2014;33:327-40.

14. Attachments

Attachment 1. Assessment protocol for heart donors (Utredningsprotokoll för donatorer)

Attachment 2. List of variables

Attachment 3. Study visit scheme

Attachment 1.

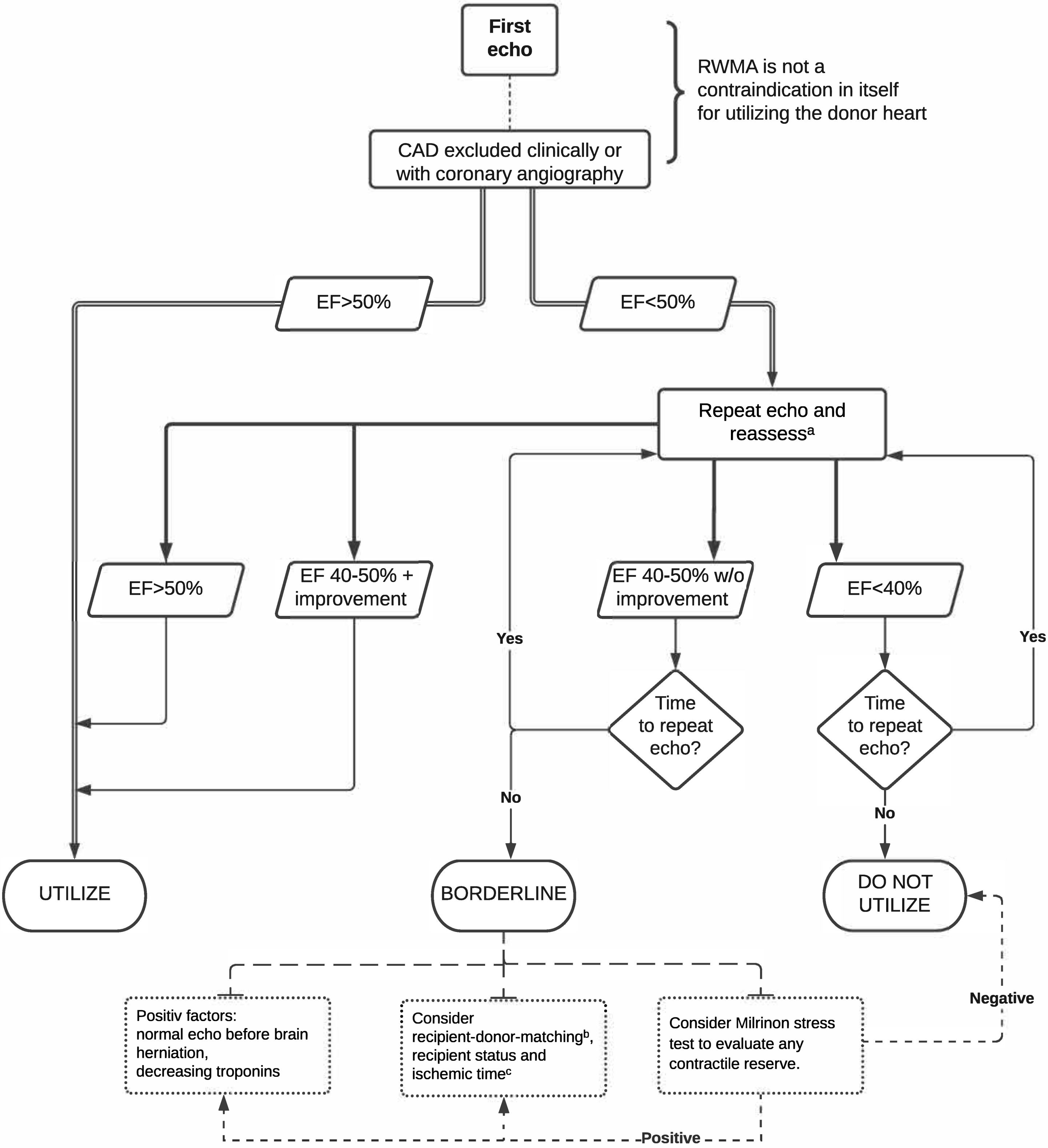

[a] Preferrably made at least six hours apart

[b] **--** Donor age:
- **-** < 45 yo preferrable
- **-** 45-50 yo if ischemic time < 4 hrs
- **-** > 55 yo only if survial benefit exceeds the decrement in early HT survival

-- Donor size:
- **-** If body weight in donor is not ≥ 30% less than recipient's, its is uniformly safe.
- \- If female donor, use with caution if donor body weight is ≥20% less than male recipient donor weight [1]

-- Donor comorbidities and cause of death

[c] Ischemic time should be ≤ 4 hrs. If greater other factors in donor and recipient should be ideal [1]

CAD: Coronary artery disease, EF: Ejection Fraction, RWMA: Regional Wall Motion Abnormalities.

[1] The International Society of Heart and Lung Transplantation
Guidelines for the care of heart transplant recipients J Heart Lung Transplant 2010;29:914 –956
