# Supplementary material for: Heart recipient outcomes following transplantation of donor hearts with impaired versus normal function: a study protocol for IMPROVED Heart, a prospective multicentre observational study
Source: BMJ Open. 2026 Jul 10;16(7):e111146. doi: 10.1136/bmjopen-2025-111146 (PMC13358299; doi:10.1136/bmjopen-2025-111146)
Supplement: online supplemental file 5 [file bmjopen-16-7-s005.pdf]

Management of the Brain-Dead Organ Donor in the Intensive Care Unit

Swedish National Guidelines

Monitoring

- Heart rate
- Arterial oxygen saturation (SaO_2)
- Mean arterial pressure (MAP) via arterial line
- Central venous pressure (CVP)
- Hourly urine output
- Temperature, preferably measured continuously via the esophagus or urinary catheter
- Nasogastric tube output
- Fluid balance every fourth hour: target –500 mL to ±0 mL (including insensible losses)
- Daily body weight
- Echocardiography as indicated according to local routines
- Advanced hemodynamic monitoring may be used when necessary (e.g., PiCCO, CVP or $ScvO_2$)

Treatment Targets

- Heart rate: 50–110 beats per minute
- SaO_2: 95–98%
- MAP: 65–100 mm Hg
- CVP: ≤10 mm Hg
- Diuresis: 0.5–1 mL/kg/h
- Hemoglobin: ≥80 g/L
- Arterial blood gas: normal pH and pCO_2; pO_2 10–13 kPa
- Blood glucose: 5–10 mmol/L
- P-Sodium: 135–150 mmol/L
- P-Potassium, calcium, and magnesium: within normal range
- Temperature: 35.5–38.0 °C

Laboratory Samples

- Blood gas analysis and measurements of glucose, sodium, and potassium initially and at least every four hours.
- Complete blood count, serum creatinine, liver enzymes (AST, ALT, ALP, bilirubin, GGT, LDH), amylase, albumin, coagulation tests (PT, APTT), and CRP initially and thereafter once daily.
- Blood typing and crossmatching.

Treatment Recommendations

Circulation

- Hypovolemia: Administer crystalloids (e.g., Ringer's acetate) and/or colloids (albumin). If diabetes insipidus with rising serum sodium levels is present, administer electrolyte-free glucose and water via a nasogastric tube. Blood products should be given as indicated, based on specific component needs.
- Hypotension (MAP <65 mm Hg): Ensure adequate fluid resuscitation. Treat peripheral vasodilatation with goal-directed vasopressor infusion. Noradrenaline or vasopressin may be used according to local practice. In cases of cardiac dysfunction requiring inotropic support, dobutamine or milrinone may be administered.
- Hypertension (MAP >100 mm Hg): Treat with labetalol. After brain death, nitroglycerin can be used.
- Tachycardia (>110 bpm): If tachycardia persists despite optimized ventilation, circulation, and temperature, β-blockers may be used provided no cardiac dysfunction is present.
- Bradycardia (<50 bpm): Treatment is unnecessary if hemodynamic stability is maintained and there are no signs of hypoperfusion. If intervention is required, use targeted isoprenaline infusion. Cholinergic antagonists are ineffective in the brain-dead donor due to loss of vagal tone but may be used prior to death.
- Hypervolemia: Avoid fluid overload due to the risk of edema and impaired organ function, particularly in the lungs and liver.
- Arrhythmia: Identify and correct the underlying cause, especially electrolyte or fluid imbalances. Administer magnesium, amiodarone, and/or a β-blocker as clinically indicated. Early electrical cardioversion may be considered if the patient is optimized with respect to fluid and electrolyte balance.

Respiration

- Lung protective ventilation: Pressure controlled ventilation.
  Plateau pressure <30 cm H_2O; PEEP 5–15 cm H_2O; driving pressure <15 cm H_2O. Tidal volume 6–8 mL/kg based on ideal body weight.
- Prevent atelectasis: Keep the lungs open with recruitment maneuvers per local protocol. Airway Pressure Release Ventilation (APRV) may be used for lung recruitment. Prone positioning may be recommended as an adjunct for atelectasis.
- Keep the lungs dry: Restrict crystalloid use in potential lung donors to reduce pulmonary edema risk. Monitor fluid balance closely. Administer diuretics as needed.
- Head-up position: Maintain a 30° head-up position throughout care.

Bronchoscopy

- Because acute illness carries a high risk of aspiration, early—and if needed, repeated—bronchoscopy is recommended. Obtain airway cultures. Due to impaired airway clearance, regular suctioning is essential. Perform bronchoscopy if secretion retention is suspected.

Hormonal Changes

- Diabetes Insipidus (urine output >4 mL/kg/h for ≥2 h): Initiate desmopressin immediately upon diagnosis. Reassess after 20 minutes; the dose may be repeated if necessary. Given its approximately 11-hour half-life, caution is advised to avoid oliguria or anuria from overdosing. Transient anuria does not preclude organ donation. Alternatively, a vasopressin infusion (<2.5 U/h) may be used, especially if hypotension is present.
- Steroids: At the time of brainstem herniation, administer methylprednisolone (Solumedrol®) 15 mg/kg as a single dose to reduce inflammation-mediated organ injury.

Metabolism

- Hyperglycemia: Common in donors due to insulin resistance and may impair graft function. Maintain blood glucose at 5–10 mmol/L using insulin and glucose infusions per local routines.

Nutrition

- Continue or initiate low-dose enteral nutrition (approximately 10 mL/h), particularly if organ-preserving treatment is expected to be prolonged. Provide ulcer prophylaxis.

Hypernatremia

- If P-Na >145 mmol/L, in addition to desmopressin or vasopressin, administer electrolyte-free glucose intravenously and tap water via nasogastric tube.

Temperature Regulation

- Target temperature: 35.5–38.0 °C. Avoid both hypo- and hyperthermia. Use active warming (e.g., warming blanket) for hypothermia. For hyperthermia, consider a cooling suit; paracetamol has limited effect in severe brain injury.

Infection

- Treat infections per culture results and current ICU protocols. After cultures, administer meropenem 0.5–1.0 g IV three times daily as ordered by the transplant surgeon, unless contraindicated.

Coagulation

- Continue thromboprophylaxis unless contraindicated.

Spinal Reflexes

- Spinal reflexes may occur due to intact spinal cord function. Administer muscle relaxants after declaration of death as needed.

Perioperative Management

- Ongoing optimization: Continue donor care per these intensive care principles until organ procurement.
- Antibiotics: Continue prescribed antibiotics until organ procurement.
- Spinal reflexes durings: Administer muscle relaxants before incision and as needed. Because spinal reflexes may cause hypertensive surges, opioids and/or volatile anesthetics are recommended.

- Blood products: Administer in consultation between the transplant surgeon and anesthesiologist.
- Anticoagulation: Heparin 400 U/kg (5000 U/mL) should be administered before perfusion catheter insertion to prevent thrombosis, as prescribed by the transplant surgeon.
- Active warming: Discontinue active warming at the time of organ procurement.

References

1. 1. McKeown DW et al. Management of the heart-beating brain-dead organ donor. Br J Anaesth. 2012;108(S1):i96–i107.
2. 2. European Committee on Organ Transplantation. Guide to the quality and safety of organs for transplantation. 8th edition, 2022.
3. 3. Society of Critical Care Medicine/American College of Chest Physicians/Association of Organ Procurement Organizations. Management of the Potential Organ Donor in the ICU. Consensus Statement 2015.
4. 4. Marik PE. Aspiration pneumonitis and aspiration pneumonia. N Engl J Med. 2001;344:665–671.
5. 5. Mascia L, Pasero D, Slutsky AS, et al. Effect of a Lung Protective Strategy for Organ Donors on Eligibility and Availability of Lungs for Transplantation: A Randomized Controlled Trial. JAMA. 2010;304(23):2620–2627.
6. 6. Parto S et al. Efficacy of recruitment maneuver for improving the brain-dead marginal lungs to ideal. Transplant Proc. 2013;45(10):3531–3533.
7. 7. Paries M et al. Benefit of a single recruitment maneuver after an apnea test for the diagnosis of brain death. Crit Care. 2012;16:R116.
8. 8. Marklin GF et al. Ventilation in the prone position improves oxygenation and results in more lungs being transplanted from organ donors with hypoxemia and atelectasis. J Heart Lung Transplant. 2021.
9. 9. Hanna K et al. Airway Pressure Release Ventilation and Successful Lung Donation. Arch Surg. 2011;146(3):325–328.
10. 10. Miñambres E et al. Lung donor treatment protocol in brain-dead donors: A multicenter study. J Heart Lung Transplant. 2015;34(6):773–780.
11. 11. Miñambres E et al. An intensive lung donor treatment protocol does not have negative influence on other grafts: A multicentre study. Eur J Cardiothorac Surg. 2016;49(6):1719–1724.
