# Supplementary material for: Heart recipient outcomes following transplantation of donor hearts with impaired versus normal function: a study protocol for IMPROVED Heart, a prospective multicentre observational study
Source: BMJ Open. 2026 Jul 10;16(7):e111146. doi: 10.1136/bmjopen-2025-111146 (PMC13358299; doi:10.1136/bmjopen-2025-111146)
Supplement: Supplementary file 6 [file bmjopen-2025-111146.reviewer_comments.pdf]

PEER REVIEW HISTORY

BMJ Open publishes all reviews undertaken for accepted manuscripts. Reviewers are asked to complete a checklist review form and are provided with free text boxes to elaborate on their assessment. These free text comments are reproduced below.

ARTICLE DETAILS

Title (Provisional)

Heart recipient outcomes following transplantation of donor hearts with impaired versus normal function - A study protocol for IMPROVED Heart, a prospective multicentre observational study

Authors

Doueh, Rana; Dellgren, Göran; Ternström, Lisa; Hård af Segerstad, Lisa; Hendén, Pia Löwhagen; Oras, Jonatan

---

VERSION 1 - REVIEW

**Reviewer**	**1**
**Name**	**Goldraich, Lívia**
**Affiliation**	**Hospital de Clínicas de Porto Alegre**
**Date**	**08-Feb-2026**
**COI**	**None**

I would like to thank the Authors for the opportunity to review this manuscript that consists of a study protocol of an interventional trial addressing the utilization of donor hearts with reduced ejection fraction. It is a prospective, non-randomized controlled multicenter, trial evaluating a standardized protocol for serial evaluations of the donor heart function to support the decision-making process. The protocol however does not directly impact the decision to proceed with transplantation, which is carried out by the attending transplant surgical team that has access to information provided by the systematic evaluations.

The study aims at prospectively addressing a relevant, controversial clinical practice of utilization of donor hearts with a depressed function that has been attributed to brain death-related mechanisms. Limitations that will be intrinsic to the study design are well reported in the protocol.

I have a few specific comments that may help to improve the strength of the manuscript.

1. Among secondary objectives, the authors described the following: “Can it be predicted early on which donor hearts will improve function during the donor process? What different mechanisms are involved in the pathogenesis of cardiac dysfunction in potential heart

donors?". How do the authors intend to address those objectives with the present study design?

2. In the study protocol for assessment of donor hearts (Figure 1), weight is used as a recommended metric for donor-recipient matching. Have the authors considered including a contemporary metric such as predicted heart mass in their recommendations?

3. Considering the different strategies that are currently available for the donor heart preservation and assessment, how do the authors plan to control for this possible confounder?

VERSION 1 - AUTHOR RESPONSE

Reviewer's comments to the author

COMMENT 1. Among secondary objectives, the authors described the following: "Can it be predicted early on which donor hearts will improve function during the donor process? What different mechanisms are involved in the pathogenesis of cardiac dysfunction in potential heart donors?". How do the authors intend to address those objectives with the present study design?
RESPONSE: We thank the reviewer for this important comment. We have clarified how these secondary objectives will be addressed within the present study design. Specifically, we have expanded the section on secondary objectives to describe how early predictors of functional recovery will be analysed using clinical, echocardiographic, and biochemical data collected during the donor process, and how underlying mechanisms will be explored through planned histological and biochemical analyses.

The change is seen in row 131 to the following:
Can it be predicted early on which donor hearts will improve function during the donor process using clinical data, echocardiographic imaging and cardiac biomarkers?
What mechanisms underlie cardiac dysfunction in potential heart donors, as determined by comparative histological and biochemical analyses of dysfunctional versus non-dysfunctional donor hearts?

Previous text:
Can it be predicted early on which donor hearts will improve function during the donor process?
What different mechanisms are involved in the pathogenesis of cardiac dysfunction in potential heart donors?

COMMENT 2. In the study protocol for assessment of donor hearts (Figure 1), weight is used as a recommended metric for donor-recipient matching. Have the authors considered including a contemporary metric such as predicted heart mass in their recommendations?

RESPONSE: In the current protocol, we have followed the recommendations outlined in the ISHLT guidelines regarding donor–recipient size matching. However, we agree that predicted

heart mass represents a contemporary metric. All relevant variables required to calculate predicted heart mass are collected within the study, enabling such analyses to be performed.

COMMENT 3. Considering the different strategies that are currently available for the donor heart preservation and assessment, how do the authors plan to control for this possible confounder?

RESPONSE: We thank the reviewer for highlighting this important point. We agree that differences in donor heart preservation and assessment strategies may act as potential confounders. In the present study, detailed data are collected according to the predefined variable list, including type of cardioplegia, preservation method, use of preservation device, and duration on device. These variables will be incorporated into the statistical analyses and adjusted for in multivariable models when evaluating associations between donor heart dysfunction, recovery, and study outcomes. The subsection *"Participant Timeline"* has been expanded to provide greater clarity regarding the prospective collection of study variables across time points, including preservation-related factors such as cardioplegia type, preservation method, use of preservation devices, and duration on device, ensuring that these factors can be appropriately accounted for in the statistical analyses. The changes are seen in row 296 to the following:

Recipient data will be collected prospectively at predefined study time points. The collected variables are selected to characterise baseline recipient status, perioperative factors, early graft function, and short- and long-term transplant outcomes.

Data will be collected and assessed at six predefined time points:

1. **At acceptance of transplantation (inclusion)** – recipient demographics, underlying diagnosis leading to transplantation, and relevant comorbidities. Baseline clinical status will include echocardiographic assessment of cardiac function, renal function, and haemodynamic measurements obtained from right heart catheterisation. Information on pre-transplant circulatory support, including inotropes and mechanical circulatory support at the time of listing will also be recorded.
2. **At the time of transplantation** – Recipient clinical status immediately prior to transplantation will be documented, including whether transplantation occurred during ongoing hospitalisation, use of mechanical circulatory assist devices and recent need for inotropic support or renal replacement therapy. Updated clinical assessments available prior to transplantation, including repeated echocardiographic assessment of cardiac function, cardiac catheterisation data and renal function measurements will also be collected. Perioperative variables including ischaemic time, cardiopulmonary bypass duration, preservation strategy, and use and duration of preservation devices.
3. **At 24 hours (±6 hours) after cross-clamp release** – assessment of early graft function including haemodynamic parameters, inotropic requirements, need for mechanical circulatory support, and evaluation of primary graft dysfunction.

4. **At discharge from the intensive care unit**– duration of intensive care, need for ongoing circulatory support, renal replacement therapy, and other early postoperative complications.
5. **At hospital discharge** – postoperative complications including surgical reinterventions and rejection episodes
6. **At one-year follow-up** – survival status, re-transplantation, need for mechanical circulatory support, haemodynamic measurements, echocardiographic assessment of graft function, cardiac catheterization with biopsies, coronary angiogram and renal function test.

Additional mortality control will be performed at three and six months after transplantation. A schematic overview of the study schedule and participant time points is shown in **Figure 2**.

Long-term complications will be collected through the Swedish National Board of Health and Welfare's Diseases, STRAX and Cause of Death Register at one, three, five, and ten years after heart transplantation.

Previous text:

Data will be collected and assessed at six predefined time points:

1. At acceptance of transplantation (inclusion).
2. At time of transplantation.
3. At 24 hours (+/- six hours) after cross-clamp release.
4. At discharge from the ICU.
5. At hospital discharge.
6. At one-year follow up.

Additional mortality control will be performed at three and six months after transplantation.

Please see figure 2 for a schematic schedule and time commitment for study participants.

Long-term complications will be collected through the Swedish National Board of Health and Welfare's Diseases, STRAX and Cause of Death Register at one, three, five, and ten years after heart transplantation.

Additional changes

- Figure 2 presenting a study overview has been revised to include the preservation method.

- All references to "Appendix A" in the manuscript have been revised to "Supplemental Material". These changes are seen in row 242, 244, 252, 332, 384 and 390.

- Citation for publication license of Figure 2 . “Study overview” updated on row 692
